# Study protocol for the sheMATTERS study (iMproving cArdiovascular healTh in new moThERS): a randomized behavioral trial assessing the effect of a self-efficacy enhancing breastfeeding intervention on postpartum blood pressure and breastfeeding continuation in women with hypertensive disorders of pregnancy

**DOI:** 10.1186/s12884-022-05325-3

**Published:** 2023-01-26

**Authors:** Natalie Dayan, Graeme Smith, Atanas Nedelchev, Haim Abenhaim, Richard Brown, Deborah Da Costa, Suhad Ali, Jesseca Perlman, Tuong-Vi Nguyen, Cindy-Lee Dennis, Wael Abdelmageed, Sonia Semenic

**Affiliations:** 1 Department of Medicine, McGill University, Montréal, Canada; 2 Department of Obstetrics and Gynecology, McGill University, Montréal, Canada; 3 Centre for Outcomes Research and Evaluation (CORE), Research Institute of the McGill University Health Centre (RI-MUHC), 5252 de Maisonneuve West, 2B.40, Montreal, Quebec H4A 3S5 Canada; 4 Department of Obstetrics and Gynecology, Queen’s University, Kingston Health Science Centre, Kingston, Canada; 5 Kingston General Hospital Research Institute (KGHRI), Kingston, Canada; 6 Department of Obstetrics and Gynecology, St. Mary’s Hospital Center, Montreal, Canada; 7 Department of Obstetrics and Gynecology, Jewish General Hospital, Montreal, Canada; 8 Department of Psychiatry, McGill University, Montreal, Canada; 9 Laurence S Bloomfield Faculty of Nursing, University of Toronto, Toronto, Canada; 10 Ingram School of Nursing, McGill University, Montreal, Canada

**Keywords:** Breastfeeding, Hypertensive disorders of pregnancy, Maternal health, Postpartum cardiovascular health, Self-efficacy

## Abstract

**Background:**

Individuals with hypertensive disorders of pregnancy (HDP) have an elevated lifetime risk of chronic hypertension, metabolic syndrome, and premature cardiovascular disease. Because breastfeeding duration and exclusivity have been associated in observational studies with improved cardiovascular health, optimizing breastfeeding in those with HDP might be an unrealized cardio-prevention approach, in particular because individuals with HDP have more breastfeeding challenges. Breastfeeding supportive interventions targeting one’s breastfeeding self-efficacy have been shown to improve breastfeeding rates.

**Methods:**

We designed an open-label, multi-center 1:1 randomized behavioral trial to test whether a previously validated self-efficacy enhancing breastfeeding intervention can improve breastfeeding duration and/or exclusivity, and lower postpartum blood pressure at 12 months. Randomization is computer-generated and stratified by site (four hospitals in Montreal, Quebec and one hospital in Kingston, Ontario; all in Canada). Included are breastfeeding participants with HDP (chronic/gestational hypertension or preeclampsia) who delivered a live singleton infant at > 34 weeks, speak English or French, and have no contraindications to breastfeeding. Informed and written consent is obtained at hospitalization for delivery or a re-admission with hypertension within 1 week of discharge. Participants assigned to the intervention group receive a breastfeeding self-efficacy-based intervention delivered by a trained lactation consultant in hospital, with continued reactive/proactive support by phone or text message for up to 6 months postpartum. Regardless of group assignment, participants are followed for self-reported outcomes, automated office blood pressure, and home blood pressure at several time points with end of follow-up at 12 months.

**Discussion:**

This study will assess whether an intensive nurse-led behavioral intervention can improve breastfeeding rates and, in turn, postpartum blood pressure – an early marker for atherosclerotic cardiovascular disease. If effective, this form of enhanced breastfeeding support, along with closer BP and metabolic surveillance, can be implemented broadly in individuals lactating after HDP.

**Trial registration:**

ClinicalTrials.gov, # NCT04580927, registered on Oct 9, 2020.

**Supplementary Information:**

The online version contains supplementary material available at 10.1186/s12884-022-05325-3.

## Background

### Long-term cardiovascular risk after hypertensive disorders of pregnancy

**Fig. 1:**
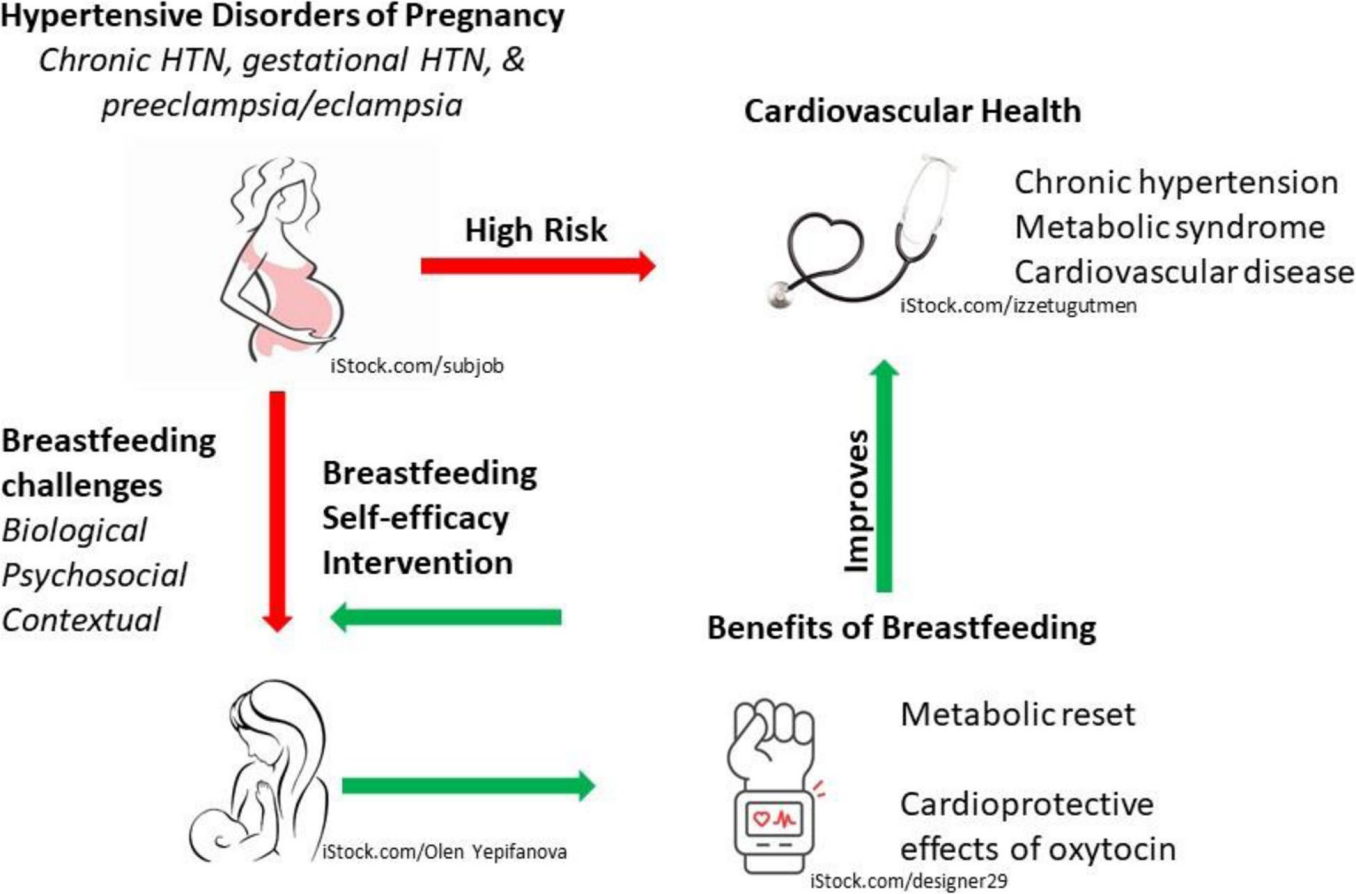
Hypothesized mechanism of cardiovascular protection of breastfeeding in women with hypertensive disorders of pregnancy. Conceptual diagram depicting associations between maternal hypertensive disorders of pregnancy and cardiovascular health, and hypothesized mediation by breastfeeding. Photos used in Fig. 1 were downloaded from iStock through a subscribed account. All photos have a Standard License, meaning we have the right to use the photos in perpetuity, and are referenced in Fig. 1

Hypertensive disorders of pregnancy (HDP), including chronic hypertension in pregnancy, gestational hypertension and preeclampsia/eclampsia [1] complicate up to 10% of pregnancies worldwide and are significant causes of severe maternal and neonatal morbidity and mortality [2]. Preeclampsia, the most severe HDP, is a complex clinical syndrome characterized by maternal hypertension and other adverse conditions associated with widespread endothelial activation and dysfunction [1, 2]. There is now overwhelming evidence from large observational studies that HDP not only impacts short-term maternal and neonatal health, but are sex-specific risk factors for premature cardiovascular disease, conferring a 3.7-fold increase in the risk of chronic hypertension, a 4.2-fold increase in risk of heart failure, a 1.8-fold increased risk of stroke, and a 2-fold increased risk of premature ischemic heart disease compared with similar individuals with normotensive pregnancies [3]. This risk is apparent at a median of 10–15 years post-affected delivery [4, 5]. Whereas cardiovascular disease risk is partly mediated by the development of hypertension and metabolic syndrome [6–9], there is evidence of persistent subclinical endothelial dysfunction for months to years following pregnancy affected by HDP, contributing to accelerated vascular impairment [10–13] (Fig. 1). The postpartum period has thus been identified as a critical opportunity to implement targeted screening and positive health interventions for those affected by HDP, and contemporary clinical practice guidelines now encourage systematic assessment of cardiovascular risk post-HDP [14, 15].

### Breastfeeding for maternal cardiovascular health

The significant benefits of breastfeeding for both infants and mothers are well-documented [16], and global recommendations for optimal infant health are to breastfeed exclusively for the first 6 months of life and to continue breastfeeding (along with appropriate complementary solid foods) for up to 2 years or beyond [17]. Although breastfeeding promotion typically focuses on infant nutrition, the optimal “dose” of breastfeeding to benefit maternal health is unknown. For example, observational data suggest that breastfeeding can lower maternal blood pressure (BP), risk of metabolic syndrome, and other markers of cardiovascular risk [18] both in the short term [19, 20] and long term [21, 22]. A recent meta-analysis including more than 1.1 million parous individuals from eight large observational studies concluded that any breastfeeding is associated with an approximately 10% lower risk of fatal and non-fatal cardiovascular disease or stroke as compared with no breastfeeding, with a progressive risk reduction for all cardiovascular disease outcomes with lifetime durations of breastfeeding [23]. Putative mechanisms include lowering of BP and metabolic syndrome [18] in the short term [19, 20] and long term [21, 22], possibly by helping to re-set the metabolic changes of pregnancy. Potential pathways for the “re-set hypothesis” include mobilization of stored fat after delivery, reestablishment of glucose homeostasis, and mobilization of lipids for milk synthesis [24, 25]. Mechanistic studies have also begun to uncover the cardiovascular role of oxytocin, the lactation hormone responsible for the milk-ejection reflex. Functional oxytocin receptors have been found in vascular beds and in the myocardium in both animals and humans, and oxytocin has been shown to have pleiotropic cardioprotective effects including systemic vasodilation and angiogenesis of smooth muscle and endothelial cells, as well as antioxidative and anti-inflammatory effects [26–28]. An inverse relationship between lactation duration and subclinical atherosclerosis (as indicated by carotid lumen thickness or aortic and coronary calcification) has also recently been identified [29–31], adding to explanations for how breastfeeding may influence BP and the development of cardiovascular events.

While few studies have specifically examined the relation between breastfeeding and postpartum BP after HDP, available evidence suggests a possible robust effect. A US study of 246 individuals with early or late-onset HDP who were 4 to 6 weeks postpartum found a mean decrease of 5.3 mmHg systolic (*p* = .03) and 3.6 mmHg diastolic BP (*p* = .04) among those who were lactating (*N* = 78) compared to non-lactating (*N* = 69) [32]. Another US study of 379 individuals with HDP demonstrated a mean decrease of 16.3 mmHg systolic/16.8 mmHg diastolic BP among those with gestational hypertension who breastfed for more than 6 months compared to no breastfeeding, adjusting for pre-pregnancy body mass index and other factors [33]. A Canadian cross-sectional analysis of postpartum BP and metabolic markers in 622 individuals with HDP and other pregnancy complications similarly found an 11% reduction in metabolic markers and a 6 mmHg decrease in median systolic BP among those who breastfed for more than 6 months compared to no breastfeeding, after adjusting for potential confounders [34]. While these observations warrant replication, the BP reductions associated with lactation exceed those seen with either weight loss [35] or antihypertensive pharmacotherapy [36].

### Breastfeeding for infant neurodevelopment after exposure to HDP

Infant brain development is a dynamic process beginning in utero that is affected by genetic, epi-genetic, and environmental factors [37]. It has been postulated that exposure to a hypertensive intrauterine environment can disrupt fetal brain development by impairing placental perfusion. A number of studies have demonstrated that fetal exposure to maternal HDP is associated with poorer neonatal neurodevelopmental compared with no such exposure [38, 39], although the mechanism for this association is not well described. Gestational age at birth and infant feeding patterns are likely important mediating factors that have not been universally accounted for in studies linking HDP with neurodevelopment [40]. However, a recent large observational study showed an increased risk of cerebral palsy, autism, epilepsy, and attention deficit hyperactivity disorder in children exposed to term preeclampsia compared with no preeclampsia [41–46], suggesting a direct effect of preeclampsia on neurodevelopment independent of gestational age. Moreover, longer breastfeeding duration has been shown to be positively associated with better motor, language and cognitive skills in infants [47]. Therefore, there is biological plausibility that breastfeeding in infants born to those with hypertension is potentially strongly protective.

### Breastfeeding rates in individuals with HDP

Despite the potential maternal and infant benefits of breastfeeding, the limited evidence available demonstrates that individuals with HDP have lower rates of breastfeeding initiation, duration and exclusivity compared to those with normotensive pregnancy [48–52]. For example, a German study of 1500 deliveries (877 with HDP) found that only 39.2% of those with HDP initiated breastfeeding compared to 48.9% of matched controls without HDP (*p* < 0.001) [48]. However, the difference in breastfeeding continuation to 3 months was smaller (37.2% in those with HDP versus 42.2% in those without), underscoring the importance of supporting successful initiation of lactation among those with HDP who intend to breastfeed [48]. A US survey of 5285 postpartum persons reported that those with HDP (*N* = 754) were significantly less likely to initiate lactation (82% vs. 87%) or to provide their infant with breastmilk for at least 8 weeks (48% vs. 55%) than those without HDP [49]. A Brazilian prospective cohort study comparing individuals with gestational hypertension (*N* = 42) to normotensive controls (*N* = 124) found that those with hypertension were significantly more likely to supplement their infants with formula during postpartum hospitalization (*p* < .00001) and to cease exclusive breastfeeding or wean by 6 months after controlling for mode of delivery, parity, and gestational age at birth, suggesting greater difficulties with maintenance of lactation over time [50].

Several potential barriers to successful breastfeeding among those with HDP have been identified, including higher rates of caesarean delivery and preterm birth; maternal-infant separation and delayed initiation of lactation; the impact of HDP medications (e.g., diuretics, magnesium sulfate) on milk production; and underlying endocrine factors (e.g., obesity, diabetes, metabolic syndrome) that may influence lactogenesis [49, 50, 53]. There may be additional under-studied biologic and psychosocial factors influencing lactation post-HDP related to the flux of reproductive hormones associated with breastfeeding (including androgens, cortisol, oxytocin and prolactin), which may moderate the risk of adverse psychological states and chronic hypertension after delivery. For example, changes in maternal androgen levels have been linked to postpartum depression [54]. Androgens have also been shown to have effects on the vascular system that are very similar to the changes observed in hypertension, including increases in vascular tone, hypercoagulability and platelet aggregation [54–58]. Psychological distress after delivery may further increase risk of hypertension by disrupting cortisol function and the hypothalamo-pituitary-adrenal (HPA) axis [59, 60]. Declines in oxytocin may impact both maternal mood and maternal insulin sensitivity and cardiovascular function [61, 62]. Finally, prolactin (the hormone responsible for milk synthesis) also appears to carry actions directly antagonistic to those of insulin, which may contribute to the metabolic changes that occur during pregnancy [63]. These hormones of lactation have also been implicated in postpartum depression [64], which may be an important mediator between HDP and low breastfeeding rates.

### Targeting breastfeeding self-efficacy to increase breastfeeding outcomes

Systematic reviews of breastfeeding supportive interventions demonstrate a significant positive impact of individual-level support on rates of breastfeeding duration and exclusivity to 6 months postpartum among healthy term infants [65, 66]. Among healthy parents, breastfeeding support interventions targeting the modifiable factor of breastfeeding self-efficacy (BSE) have been associated, in particular, with increased rates of breastfeeding duration and exclusivity [67, 68]. Derived from Bandura’s social cognitive theory, breastfeeding self-efficacy refers to a persons’ confidence in their perceived ability to breastfeed their infant, and is influenced by four main sources of information (performance accomplishments, vicarious learning, verbal persuasion, and physiological/affective states) [69]. Co-investigator C-L Dennis developed and validated the 33-item Breastfeeding Self-Efficacy Scale (BSES) [70] and 14-item BSES-Short Form [71], which has also been adapted into a validated measure of BSE among parents with ill or preterm infants [72]. BSE has long been identified as a potent predictor of breastfeeding duration and exclusivity in diverse populations [73–77]. A meta-analysis of interventions to improve BSE found that participants in intervention groups had significantly higher BSE (scoring 4.86 points higher at 2 months postpartum) and that the odds of exclusive breastfeeding at 2 months increased by 10% in the intervention groups for each 1-point increase in mean BSE score between intervention and control groups [67]. Moreover, BSE-focused interventions were most successful when delivered in both hospital and community settings using multiple contact points [67]. Although several studies have underscored the need for enhanced breastfeeding support for those with HDP [51–53], interventions designed to increase breastfeeding self-efficacy have not yet been tested specifically in this population. Individualized support targeting self-efficacy may help those with HDP overcome their unique breastfeeding challenges, as well as enhance parental engagement in other lifestyle interventions (e.g., weight loss, exercise, healthy eating) which have been shown to decrease cardiovascular risk post-HDP [78].

### Supportive pilot data

Feasibility and acceptability of the randomized control behavioral trial (RCT) procedures and the breastfeeding self-efficacy intervention (BSEI) were determined in a single-center pilot RCT conducted between February 2019 and November 2020, entitled Breastfeeding and blood Pressure patterns in MOthers with recent hypertensive coMplications of pregnancy (BP-MOM study, ISRCTN.com, # 18352227, registered on Jan 29, 2019). Of 183 individuals with HDP screened for study eligibility, 90 met the inclusion criteria and were approached. Of these, 45 agreed to participate, completed baseline measures and were randomized to usual care (*N* = 21) or the BSEI (*N* = 24) [79]. Follow-up to the 6-month clinic appointment was completed by 37 (82%) participants. Follow-up to 12 months was interrupted by the first wave of the COVID-19 pandemic, and was conducted predominately remotely due to public health restrictions (*N* = 27, 80% completed remote and *N* = 7, 20% completed in person 12-month follow-up).

**Fig. 2:**
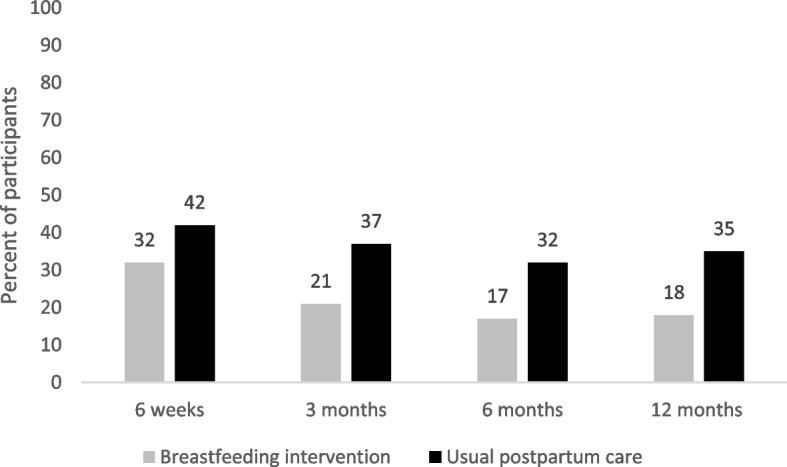
Elevated Blood Pressure or Use of Anti Hypertensive Medication in Post Partum Women with HDP. This figure shows mean participant blood pressures according to study allocation group (breast feeding self-efficacy based intervention or usual postpartum care) at each of the study time points from the feasibility study “Breastfeeding and Blood pressure patterns in MOthers with hypertensive coMplications of pregnancy (BP-MOM)”. Elevated blood pressure was defined as average automated office blood pressure > 140/90 mmHg or average home blood pressure > 135/85 mmHg

Mean systolic BP was lower at all time-points in the intervention group than the usual postpartum care group (Table 1), as was number with BP in the hypertensive range, and number requiring anti-hypertensive therapy (Fig. 2).

**Table 1:** BP-MOM mean systolic and diastolic blood pressure at each time point in postpartum women with HDP

	Total	Breastfeeding Intervention	Usual Postpartum Care	*p*-value
N	45	24	21	
Baseline systolic (m, sd)	129 (11.3)	127 (11.8)	132 (10.5)	.29
Baseline diastolic (m, sd)	82 (8.9)	80 (10.3)	85 (6.0)	.11
N	36	18	18	
3-month systolic (m, sd)	115 (12.1)	115 (11.9)	116 (12.5)	.77
3-month diastolic (m, sd)	74 (8.4)	72 (8.3)	75 (8.4)	.29
N^a^	31	15	16	
6-month systolic (m, sd)	113 (11)	112 (12.3)	114 (10)	.70
6-month diastolic (m, sd)	72 (10.2)	71 (11.7)	73 (8.7)	.56
N^b^	29	13	16	
12-month systolic (m, sd)	118 (13)	117 (10.1)	119 (15.3)	.75
12-month diastolic (m, sd)	76 (9.6)	77 (9.8)	76 (9.7)	.61

^a^ Data at 6-month time point does not reflect full sample size as in-person visits were limited due to the COVID-19 pandemic. ^b^ Blood pressure values at 12-months were derived from both home blood pressure measurements (self-reported) and in person automated office blood pressure measurements due to restrictions for in-person visits during the COVID-19 pandemic

In addition, mean breastfeeding self-efficacy scores (out of a total of 70) were higher in the intervention compared to the control group at 6 weeks (54.7 +/− 9.5 vs. 46.1 *+/−* 13.1), 3 months (55.3 +/− 13.9 vs. 46.3 +/− 15.9) and 6 months (57.2+/− 12.9 vs. 50.3 +/− 18.3). Compared with participants in the usual care group, those who received the BSEI were more likely to be breastfeeding exclusively at 6 weeks (71.4% vs. 44.4%) and 3 months (72% vs. 37%), and to continue breastfeeding to at least 6 months (90% vs. 58%).

The vast majority of participants assigned to the intervention group reported good or very good overall satisfaction with the BSEI at both 3 months (95%, *N* = 19) and 6 months (94%, *N* = 17). Given the positive outcomes and high acceptability of the BSEI, a recruitment rate of 50% within a high-risk obstetrical population, and participant retention of 82% at 6 months, our research team’s steering committee agreed that proceeding with a larger trial was warranted with a protocol adapted for COVID-19 pandemic contingencies (e.g., incorporating options for remote recruitment and measurements).

### Summary and rationale

Although individuals with HDP are at substantial risk for chronic hypertension, metabolic syndrome, and premature cardiovascular disease (CVD) and may benefit in particular from the cardiometabolic effects of breastfeeding, they face additional lactation challenges (Fig. 1). BSE-based supportive interventions have shown promise in improving breastfeeding rates, and our single-centre pilot data further supported a metabolic trend in the parent. There are existing knowledge gaps as to the optimal strategy to optimize breastfeeding and improve postpartum cardiometabolic health in this population, which we hope to address with the sheMATTERS study.

## Methods

### Study objectives and hypotheses of sheMATTERS

**Fig. 3:**
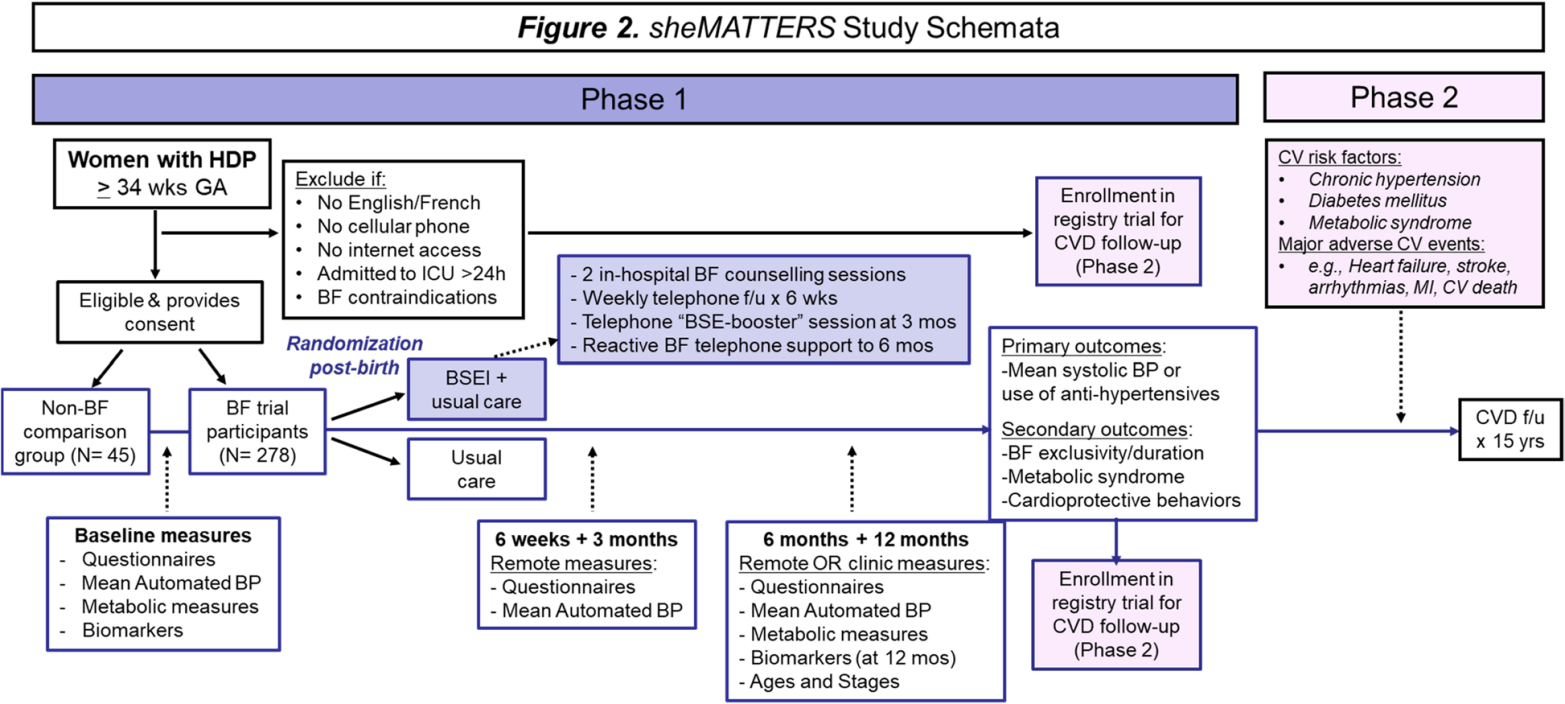
sheMATTERS Study Schemata. Schema of the sheMATTERS randomized behavioral trial design, additional comparison group, and long-term pragmatic cohort

The overall aim of the sheMATTERS (iMproving cArdiovascular healTh in new moThERS) trial is to assess the impact of an individualized BSEI on postpartum blood pressure and breastfeeding rates among patients with HDP who initiated breastfeeding. Longer-term (15-year) post-trial follow-up of study participants will additionally be conducted, to observe whether breastfeeding support, and breastfeeding duration/intensity helps to lower the incidence of chronic hypertension or other cardiovascular risk factors, as well as heart disease, stroke, and other long-term health issues among those with HDP (Fig. 3).

The primary study objective is to assess whether the BSEI will result in lower systolic and/or diastolic BP, or a lower need for antihypertensive therapy at 12 months postpartum. Secondary study objectives are to 1) assess whether the BSEI will result in higher rates of exclusive and any breastfeeding at 6 months, as well as continued breastfeeding to 12 months; 2) assess whether the BSEI will decrease the rate of having metabolic syndrome at 12 months postpartum; 3) explore maternal blood pressure trajectories over time according to ‘dose’ of breastfeeding; 4) evaluate whether those who breastfeed for longer are more likely to engage in other cardiovascular protective behaviors, such as weight reduction, healthy eating, and higher levels of physical activity at 6 and 12 months postpartum; 5) explore the biological and psychosocial determinants of breastfeeding behavior to 12 months postpartum among those with HDP; and 6) collect bio-samples to measure key inflammatory and angiogenic factors from participants who recently had HDP to advance our understanding of the different phenotypes of these individuals and their associated cardio-metabolic risk in the short and long-term.

### sheMATTERS trial hypotheses

The primary study hypotheses are that 1) individuals with HDP who receive the BSEI will have lower BP (approximately 5 mmHg systolic and/or 2 mmHg diastolic) compared to those who do not breastfeed, and 2) individuals with HDP who receive the BSEI will have a lower demand for antihypertensive treatment at 12 months postpartum compared with those who receive usual postpartum care. The secondary hypotheses are that:

- there will be a modest (10–20%) relative benefit in breastfeeding outcomes among participants with HDP who receive the BSEI compared to those who receive usual postpartum care;
- there will be a lower rate of metabolic syndrome in participants with HDP who receive the BSEI compared to those who receive usual postpartum care, and a greater difference when compared to those who do not breastfeed;
- rates of breastfeeding duration and exclusivity will be correlated with a greater tendency towards more cardioprotective behaviors over 12 months postpartum;
- infant neurodevelopment will be positively correlated with longer duration of any breastfeeding;
- severity of HDP, obesity, markers of inflammation, as well as education level and psychological symptoms at birth will be correlated with breastfeeding duration and exclusivity over 12 months postpartum.

### Study design and setting

The sheMATTERS trial is a multicenter, open-label, RCT conducted at four publicly funded, university-affiliated teaching hospitals in Canada (three located in Montreal, Quebec and one in Kingston, Ontario) with ~ 12,000 annual deliveries combined. The trial is registered on ClinicalTrials.gov (identifier # NCT04580927; see Additional file 4: Appendix 1 for Trial Registration Data Set). The coordinating center, the Research Institute of the McGill University Health Center (RI-MUHC), is responsible for overall data management, monitoring, and communication among all sites, and general oversight of the research project.

### Participants and recruitment

We are recruiting eligible postpartum (*N* = 278) breastfeeding parents with HDP into the RCT to assess the effect of the BSEI on BP and breastfeeding outcomes. In addition, we are recruiting (*N* = 45) non-breastfeeding parents with HDP into a non-randomized observational arm of the study, serving as a comparison group. Inclusion criteria include: having a diagnosis of HDP, as per the 2018 International Society of Hypertension in Pregnancy (ISSHP) classifications [80], age ≥ 18 years, singleton live birth delivered at ≥34 weeks gestation, speaks and understands English or French, has cellular phone and internet access, has a valid health insurance card at time of recruitment, and (for those in the randomized arm), intends to breastfeed and initiates breastfeeding or milk expression prior to hospital discharge. Exclusion criteria include: maternal intensive care unit admission lasting > 24 hours, severe or uncontrolled psychiatric illness that would preclude active engagement in the study, active COVID-19 infection at time of postpartum hospitalization, or previous participation in the trial’s pilot study. In addition, breastfeeding individuals are excluded from the randomized arm if they or their infant have any absolute contraindication to breastfeeding (e.g., HTLV-1 infection; galactosemia) or conditions that may interfere with successful initiation of breastfeeding (e.g., history of breast reduction surgery; infant with cleft palate).

Potential participants are identified and recruited from the hospitals’ antenatal, intrapartum or postpartum units by trained research assistants. Participants may also be referred antenatally to the research team by their obstetrical care provider or self-refer in response to recruitment posters and flyers distributed in the participating centers. The research staff meet with interested participants in person or via telephone to assess eligibility, and obtain informed verbal and written consent (see Additional file 5: Appendix 2). All eligible persons, including those who decline participation in the RCT or observational arms of the study, are additionally invited to participate in an independent long-term passive follow-up study via electronic health record data linkage.

All participants have the ability to withdraw at any time before study completion. Participation may also be stopped by the research team if a second pregnancy occurs during the 12-months study, or if it is in the best interest of participants (i.e., new medication, participant’s mental health condition). Data collected until then is kept guaranteeing the integrity of the study conduct but will not be used for analysis.

### Randomization procedures

After giving birth, all eligible and consenting postpartum individuals who are planning to breastfeed are enrolled in the RCT and randomly allocated by the research assistant to either the control group (usual in-hospital and community postpartum care only) or intervention group (usual in-hospital and community postpartum care plus the BSEI with an assigned lactation specialist). A usual care group as a primary comparison arm is best suited to assess the effectiveness of a behavioral intervention. A central random allocation sequence is computer-generated (via REDCap) stratified by study site, with an allocation ratio of 1:1 using random block sizes of 2, 4 and 6. Allocation is concealed to all research team members until randomization. The research assistant informs the participants of their group allocation immediately after they’ve completed their baseline measures. Although the RCT participants cannot be blinded, data oversight and analysis will be performed by research personnel blinded to group allocation.

### Usual postpartum care and non-breastfeeding comparison groups

All study participants, regardless of intervention status, receive usual in-hospital and community postpartum care. In Quebec and Ontario, length of postpartum hospitalization for those with HDP is on average 24 hours longer than the standard stay of 24–36 hours following uncomplicated vaginal birth, and 72 hours following cesarean section. In Quebec, community-based public health nurses routinely contact all postpartum parents via telephone within 72 hours of hospital discharge. In-hospital and community-based perinatal nurses have at least basic training in breastfeeding assessment and support. Other breastfeeding resources that may be available to all participants include access to hospital-based lactation consultants, government-issued booklets on infant care, resource lists for community-based breastfeeding support and on-line breastfeeding support websites. In Ontario, education sessions are available for new parents to help with newborn care and feeding. In addition, a public health nurse may visit and offer breastfeeding support, and teach parents how to care for themselves and their baby. Home and Community Support can also provide assistance and resources. As part of usual clinical care post-HDP, all study participants are also scheduled for an in-person appointment at the Maternal Cardiovascular Health Clinic (Montreal), or the Maternal Health Clinic (Kingston) at 6- and 12-months postpartum.

As a token of appreciation for their participation in the study, participants receive a gift bag valued at $20.00 CDN containing samples of newborn products from collaborating suppliers immediately after informed consent has been obtained. In addition, at the end of the study, participants receive a gift card valued at $20.00 CDN. In recognition of their time and effort, participants are compensated for parking or transportation expenses in the amount of $10.00 CDN per in-person study visit, for a total of two visits. Funding for this expense is covered by Dr. Dayan’s Early Career Professorship in Women’s Heart Health from the Heart and Stroke Foundation of Canada (Fund #252076).

### Intervention group

In addition to usual care and anything sought out in the community, participants allocated to the BSEI receive individualized breastfeeding support from a trained research lactation consultant. The BSEI consists of two in-hospital, one-on-one BSE-enhancing sessions prior to postpartum discharge, weekly proactive telephone follow-up calls from the lactation consultant for the first 6 weeks post-hospital discharge, and a telephone BSE-enhancing “booster” session at 3 months. In addition, the lactation consultant is available via phone or text for participant-initiated retroactive breastfeeding support for up to 6 months postpartum (the recommended duration of exclusive breastfeeding) (Fig. 3).

The BSE-enhancing sessions were adapted from a BSEI developed by co-investigator C-L Dennis that has been pilot-tested [81] and recently trialed with almost 1000 first-time parents in Canada (ISRCN.com, #85493925). The sessions last on average 60 minutes and follow a standardized format of 1) assessment of the participant’s breastfeeding goals, general physiologic state (e.g., pain, fatigue), and level of BSE using the Breastfeeding Self-Efficacy Short Form (BSES-SF) [71]_,_ 2) observation of the participant’s breastfeeding or milk expression attempts (when possible), and 3) implementation of tailored BSE-enhancing breastfeeding management strategies based on the four sources of self-efficacy information [69]. For example, if the participant indicates low confidence for the BSES-SF item: *“I can always determine that my baby is getting enough milk”,* individualized interventions may include pointing out observable cues of effective infant latch and milk transfer at the breast (performance accomplishments), praising successful breastfeeding attempts (verbal persuasion), and using visual aids to describe newborn milk-volume requirements during the first days of life (vicarious learning). For participants with infants admitted to the neonatal intensive care unit (NICU), the BSE assessment is conducted using the BSE scale adapted for parents with ill or preterm infants [72]. The first in-hospital BSE-enhancing session occurs as soon as possible following randomization to the BSEI. Immediately upon allocation of a participant to the BSEI, the study’s lactation consultant is notified and contacts the participant via telephone to arrange a time for the first BSE-enhancing session (ideally to coincide with an infant feeding). The session may be conducted in the participant’s postpartum hospital room or at the infant’s bedside in the NICU, as needed. A second BSE-enhancing session is scheduled prior to the participant’s hospital discharge if time permits, to continue the breastfeeding management strategies and reinforce improved performances. If the lactation consultant is restricted from visiting the hospital units due to COVID-19 infection control procedures, the in-hospital BSE-enhancing sessions will be conducted remotely by telephone and/or videoconferencing using the research team’s confidential ZOOM™ license. The 3-month telephone BSE-enhancing “booster” session is conducted by telephone, with the option of videoconferencing via ZOOM™ if observation of an infant feeding session is needed for the breastfeeding assessment. The goal of the 3-month session is to provide BSE-enhancing strategies for any new or ongoing breastfeeding issues and to boost participants' confidence and commitment to sustain exclusive breastfeeding up to 6 months.

As the first 6 weeks postpartum has been identified as a critical time-period for the establishment of successful lactation [82], the first follow-up telephone call from the lactation consultant is scheduled for 7 days post-discharge, and then once weekly thereafter for 6 weeks. Up to five attempts to contact the participant via either phone or text is made for each follow-up call. These proactive calls last on average 30 minutes and follow a standardized format of 1) assessment of the participant’s breastfeeding status and physiological state, 2) review of lactation progress since last contact, and 3) implementation of BSE-enhancing breastfeeding management strategies tailored to the participant’s ongoing issues or concerns. For the retroactive telephone breastfeeding support, BSEI participants are provided with the lactation consultant’s cell phone number and encouraged to call or text for any additional breastfeeding related-questions or support needed, up to 6 months postpartum.

### Study outcomes

Our primary study endpoint is average systolic and/or diastolic BP, in mmHg, measured by standard recommended technique using validated automated office blood pressure devices. In the case of home blood pressure, an average of four daily measurements over a one-week period is used, after discarding the first day of measurement. Blood pressure data is collected at baseline, 6 weeks, 3 months, 6 months, and 12 months postpartum with the primary outcome at 12 months with repeat blood pressure and other cardiometabolic markers, as well as novel biomarkers. The purpose of the latter measurement is a secondary aim of sheMATTERS, which is enhanced cardiovascular phenotyping of those with HDP.

Our secondary study endpoint includes 1) elevated blood pressure (displayed automated mean blood pressure > 135 mmHg systolic and/or > 85 mmHg diastolic) or use of/increase in previous use of antihypertensive drug therapy, and 2) the presence of metabolic syndrome [83]. An additional secondary endpoint is systolic and/or diastolic blood pressure at 6months postpartum.

Breastfeeding outcomes include the duration of exclusive breastfeeding (in weeks), total breastfeeding duration (in weeks), the proportion of participants exclusively breastfeeding at 6 months, and the proportion of participants continuing any breastfeeding at 6 months and 12 months. Given high rates of in-hospital formula supplementation of breastfeeding infants born to persons with HDP [50], we have defined exclusive breastfeeding as the provision of nothing but breastmilk (either at the breast or via milk expression) after the first 7 days of life.

### Data collection

The study procedures are shown in Table 2. Study data is collected at baseline (i.e., the delivery hospitalization), 6 weeks, 3 months, 6 months, and 12 months postpartum. All study data is entered by the participants or research staff directly into REDCap, a secure, encrypted web-based electronic data capturing system (www.projectredcap.org). In hospital, participants complete baseline self-report questionnaires either electronically on their cell phone or a Samsung Galaxy Tablet (provided by the research assistant), or on paper. For the subsequent time points, participants are sent electronic links and email reminders to complete their follow-up self-report questionnaires online via REDCap. The research lactation consultants enter all data and clinical notes related to BSEI assessments and interventions directly into the BSEI Activity Logs in REDCap, and breastfeeding-related outcome data are collected via self-report using an infant feeding questionnaire adapted from McQueen et al’s BSE-enhancing intervention pilot trial [81].

**Table 2:** sheMATTERS study procedures

Time Point	2	3	4	5	6
	Screening & Baseline	Six Week	Three Month	Six Month	Twelve Month
***Maternal Data***					
**Questionnaires**					
Medical and obstetrical history	**X**				
Maternal characteristics questionnaire	**X**	**X**	**X**	**X**	**X**
Maternal concomitant medications	**X**	**X**	**X**	**X**	**X**
Breastfeeding Self-efficacy Scale (randomized participants only)	**X**	**X**	**X**	**X**	**X**
Healthy Eating Score Questionnaire	**X**		**X**	**X**	**X**
Edinburgh Postnatal Depression Scale	**X**		**X**	**X**	**X**
STAIT/TRAIT Anxiety Scale	**X**		**X**	**X**	**X**
Infant Feeding Questionnaire	**X**	**X**	**X**	**X**	**X**
Maternal Satisfaction Questionnaire (intervention participants only)			**X**	**X**	
**Clinical measures**					
Vital signs (heart rate, respiratory rate, temperature)	**X**				
Blood pressure (SunTech CT40 BP Machine)	**X**			**X**	**X**
Blood pressure (A & D Medical Self-Monitoring BP Machine UA-651)	**X**	**X**	**X**	**X**	**X**
Height, weight	**X**			**X**	**X**
Hip and waist circumference				**X**	**X**
**Biological samples**					
Blood draw for Clinical Laboratory Tests (biochemistry, lipid profile, thyroid profile, microalbumin profile)	**X**			**X**	**X**
Additional 4 ml blood draw for Biomarker Analysis (endothelial and inflammatory markers, circulating plasma micro-RNA)	**X**				**X**
Random Urine, Routine Urinalysis				**X**	**X**
***Infant Data***					
Infant medical data	**X**		**X**	**X**	**X**
Ages and Stages Questionnaire – 3rd Edition					**X**

To collect socio-demographic and maternal health information, participants complete the Maternal Characteristic Questionnaire at each time point (see Additional file 1). We are using the self-report Edinburgh Postnatal Depression Scale (EPDS) [84] and State-Trait Anxiety Inventory (STAI) [85] to assess postpartum depression and anxiety, respectively, at baseline, 3 months, 6 months, and 12 months postpartum. Previous norms indicate that total scores of 10 or above on the EPDS and 50 or above on the STAI are consistent with clinically relevant depression and anxiety, respectively [86]. The Breastfeeding Self-Efficacy Scale (BSES), a validated 33-item questionnaire, captures information on the participant’s breastfeeding techniques, beliefs and attitudes using a 5-point Likert scale for each item [87]. Breastfeeding outcomes are captured by the Infant Feeding Questionnaire (IFQ), which collects detailed information on duration and exclusivity of breastfeeding, reason for supplements, satisfaction, and any difficulties [88]. The BSES and IFQ are sent to participants at every time point or until the cessation of breastfeeding is reported. For the intervention group, an evaluation of participants’ satisfaction with the intervention using the modified satisfaction questionnaire from Dennis’s WISE trial, is sent at 3 months and 6 months. Self-reported physical activity is collected at 3 months, 6 months and 12 months using the validated International Pregnancy Physical Activity Questionnaire (PPAQ) [89]. The Healthy Eating Score, a newly developed composite score assessing adherence to the Canada Food Guide [90], is being used to assess quality and variety of the diet at baseline, 3 months, 6 months, and 12 months postpartum. Lastly, at 12 months, parents complete the Ages and Stages Questionnaire, Third Edition (ASQ-3) [91], which has shown to be a reliable and valid instrument for determining the need for further infant developmental evaluation [92, 93].

Maternal and infant medical data is collected by research assistants via medical records reviews at baseline and recorded on our Maternal or Infant Clinical Measures Form (CMF). At subsequent time points, the Maternal CMF documents current health status and cardiovascular health, and the Infant CMF documents the child’s health and growth (see Additional files 2 for Maternal CMFs and 3 for Infant CMFs). Clinical measures are obtained by research staff during postpartum hospitalization (BP and weight) and clinic follow-up visits at 6 months and 12 months (BP, weight, hip and waist circumference). BP is measured using the same validated automated office blood pressure device (SunTech CT40 BP machine) at each site. All members of the research team are trained in proper technique of blood pressure measurement, as recommended by Hypertension Canada (https://guidelines.hypertension.ca/). Briefly, blood pressure measurement is taken with appropriately sized cuffs, in the seated position with legs uncrossed and arms positioned at the level of the heart, in a quiet room. Five measurements are taken with a one-minute interval between each, and an average of measurements is displayed by the machine.

At baseline, all participants are loaned a home BP monitor (LifeSource® A&D Medical) and provided with a web-based, Bluetooth-enabled, encrypted smartphone application (Sphygmo™) to enable remote transmission of their BP measures to the research team. A member of the research team provides instructions on how to measure and transmit their BP at home (ideally twice in morning, twice in the evening daily for 1 week prior to each scheduled study follow-up). The research team assists with installation of the mobile app prior to hospital discharge. Participants receive an automatic email about 10 days prior to the data collection time-point as a reminder to take their blood pressure and complete the online questionnaires. In addition, text messages via a mobile app are sent to participants a few days after the original email as a second reminder to take their blood pressure. The study coordinator tracks adherence of BP monitoring through the Sphygmo™ app as an end-user.

Blood and urine samples are collected for a routine metabolic panel at 6 months and 12 months, as part of usual postpartum care of those with HDP. A comprehensive metabolic panel is conducted in accordance with local laboratory protocols, and analyzes the following: glycosylated hemoglobin, fasting glucose, lipid profile [includes total cholesterol, non-HDL cholesterol, triglycerides, calculated LDL cholesterol], urine analysis, urine microalbumin to creatinine quantification, serum creatinine, serum electrolytes [sodium, potassium, chloride, bicarbonate, calcium], apolipoprotein-B. At baseline and at 12 months postpartum, we also collect an additional four ml of blood to process for analysis of HDP biomarkers (angiogenic factors, inflammatory factors, circulating plasma micro-RNA). Samples for biomarker analysis (serum and plasma) are kept frozen (− 80 °C) at a local freezer until the end of the recruitment period and will then be shipped on dry ice in batches directly to the laboratory for analysis (see Additional file 6: Appendix 3).

If participants are not able to attend the follow-up clinic visits in person, clinical data can be collected remotely. The study physician will schedule a meeting with the participant via ZOOM™ to conduct the post-HDP follow-up assessment. All participants are provided with a measuring tape upon enrollment for self-measurement of waist and hip circumference, and participants can self-report their weight using their own scales. BP measurements will be obtained remotely using the participants’ home BP monitoring machine loaned to them at recruitment. The participants will also be emailed a requisition for biospecimen collection (blood, urine) that can be conducted at their local walk-in health centers.

Finally, participants who consent will also participate in pragmatic long-term follow-up via data linkage to ascertain cardiovascular outcomes (Fig. 3).

### Sample size calculation

In order to detect a 5 mmHg between-group differences in postpartum blood pressure and in breastfeeding rates, we are aiming to recruit an approximate of 323 participants (after accounting for a 30% loss to follow-up). Around 278 of these participants (86%) will be randomized to either the breastfeeding intervention group or breastfeeding control group, and 45 participants (14%) who will not be breastfeeding will serve as a comparison group in the observational arm of the study. Accounting for annual delivery rates at each of the study sites and assuming that ~ 8% of deliveries are affected by HDP at all sites, and 70% of those will initiate breastfeeding (an average of general Canadian population initiation rate of ~ 90%, German HDP population rate of 40%, and United States rate of 82% in those with HDP) [48, 94] we anticipated reaching our recruitment target over a two-year period, without accounting for additional recruitment delays due to the pandemic.

### Statistical analysis

Descriptive statistics will be used to characterize the study population. We will compare baseline characteristics in the experimental study arms to assess randomization effectiveness. We will assume two-sample tests such as chi-square tests, t-tests, and non-parametric tests as appropriate to assess between-group differences in various characteristics and outcome measurements. They include proportion on antihypertensive drug therapy, proportion of EBF at 6 months, total breastfeeding duration in weeks, total EBF in weeks, metabolic syndrome, as well as differences in physical activity and dietary patterns. Differences in the primary outcome between the intervention and control groups on study outcomes will be assessed using an intent-to-treat approach. To examine the effect of BSEI on systolic and/or diastolic BP at 12 months, we will use generalized linear regression (GLM) to model for continuous BP readings and the Generalized Estimating Equation (GEE) analyses using logistic regression (reduction of 5 mm in SBP from the baseline vs. no change). We will calculate beta estimates with 95% confidence intervals (CIs) after adjusting for any observed between group differences despite randomization. Given that we plan to recruit non-breastfeeding parents (*N* = 45), we will model breastfeeding first as a continuous exposure variable (number of weeks) with systolic BP as the outcome in a multivariable linear regression model. We will also evaluate the dose-response of breastfeeding categories (i.e., none, < 3 months, < 6 months, > 6 months) to determine systolic and diastolic BP trajectories. In a sensitivity analysis, we will use different combinations of systolic blood pressure and diastolic blood pressure, and delta change in BP from baseline will be assessed. Breastfeeding duration will be divided into quintiles/quartiles, and those will be correlated with a series of BP measures. We will also perform a survival analysis modeling time to cessation of exclusive breastfeeding in both study groups. The model will incorporate interval censoring and compare differences between groups controlling for variables of interest, such as baseline self-efficacy score, maternal education, income, and age. Kaplan-Meir curves and cox-regression will be used to address these aspects.

For secondary analyses, the statistical method to compare groups will depend on the distribution of the outcome variables in question. Mediation effects will be assessed using stratified analyses according to pre-defined clinically relevant characteristics such as parity (1 vs. > 1) and gestational age at delivery (34–37 weeks vs. > 37 weeks). Site-specific, sub-group analyses will be performed to know whether there are differences in the study populations. Statistical analysis will be performed using SAS 9.4 version, performed by the biostatistical unit at the primary study site, guided and overseen by the principal investigators.

### Fidelity of the protocol

The research lactation consultants have received in-depth training to deliver and document the BSEI. To ensure consistency of intervention approach across the sites and monitor for potential slippage in intervention quality over time, up to 6 in-hospital BSEI intervention sessions per lactation consultant will be audio-recorded (with signed consent from the participant) and scored for intervention quality by co-PI Dr. Semenic.

### Data management and study oversight

All study procedures follow standard operating procedures set forth by Good Clinical Practice Guidelines and overseen by a project manager, with relevant tasks delegated to research assistants and recorded in a task delegation log.

Data taken from parents and infants medical charts, as well as Sphygmo™, is entered directly into REDCap by a member of the research team, and is later verified by a second team member.

Participants’ self-reported data is entered directly into REDCap by the participant. The research team is notified via email once all questionnaires are completed at each time point by a participant, at which point a member of the team verifies completion and composite scores of all questionnaires. If one or more items are left blank, the staff will email the remaining question(s) to the participant with the possible responses (if applicable) and asks for their response(s). This is done to minimize missing data. There is no formal data monitoring committee, as the intervention being provided to participants is behavioral so there is minimal risk of harm. Site PI’s have access to the raw data collected from participants recruited at their institution. The RI-MUHC, as coordinating center, has access to all raw data. The RI-MUHC can also decide to audit the study at any time. The study is subject to ongoing review and approval from Research Ethics Boards (REB) with institutional jurisdiction. In Quebec, based on the mechanism set forth by the MSSS for multicentre studies, the REB responsible for protocol revisions and approval is the RI-MUHC REB.

### Safety reporting

#### Description of the provisions for dealing with medical complications

Medically related complications applicable to the study are complications affiliated with venipuncture and venous blood withdrawal. For this reason, blood samples are being drawn by a qualified laboratory technician, nurse, or a medical doctor with training and experience in performing such procedure.

Pregnancy while participating in the study is a possibility. Interval pregnancy is recorded and marks the end of active study follow-up, but the treatment in this study does not pose any risk to subsequent pregnancies.

#### Methods for recording adverse events (AE)

If any side effects or changes in participants’ health become apparent during this trial, which are presently unexpected, these will be reported to the individual Principal Investigator or the clinic staff to be evaluated as potential AEs. The PI will assess any untoward changes as AEs based on their clinical judgment, as well as designate causality and relationship of the AEs to the study in the participant’s records.

#### Adverse events and serious adverse events reporting

All observed or volunteered adverse events regardless of study group will be recorded on the Adverse Event page(s) of the electronic Case Report Form (eCRF) (Clinical Measures Form Mother, completed by the research Staff). Events involving adverse drug reactions to prescribed hypertensive and/or other medications during the study, illnesses with onset during the study or exacerbations of pre-existing illness will be recorded on the AE eCRF. Objective test findings (e.g., abnormal laboratory test results) will also be recorded.

For all adverse events, the site PI must pursue and obtain adequate information to determine the outcome of the adverse event and to assess whether it meets the criteria for classification as a Serious Adverse Event (SAE) requiring immediate action. Any unexpected or serious adverse event will be reported to the REB in compliance with institutional policy.

#### Reporting mental health conditions

There is a high prevalence of postpartum mood disorders (e.g., postpartum anxiety or depression) in the general population, and our study involves parents with health conditions who may face additional challenges post-birth. As we observed in our BP-MOM feasibility study, we may identify parents with higher than normal scores on the psychological measures for anxiety (STAI) and depression (EPDS). A member of the research team reviews the participants’ STAI and EPDS scores shortly after they are completed by the participant. Elevated scores on the EPDS or STAI do not by themselves constitute an adverse event but will be used as needed to trigger the standard of care mental health follow-up procedure of the involved site.

Mental health referral will also be triggered if the participant’s answer to the EPDS question # 10 (“The thought of harming myself has occurred to me”) is “Sometimes” or “Quite often”, regardless of EPDS total score. Visits to psychiatric emergency wards, hospitalization, or suicide attempt or completion will be considered Adverse events and/or Serious Adverse Events.

### Privacy and confidentiality

Measures in place are consistent with legal requirements to protect privacy and confidentiality of stored study data, length of data retention and the eventual disposal of collected biological specimens. To enhance anonymity and confidentiality, individual-level data is coded, de-identified, and kept separately from all other study data in a locked cabinet at all participating sites. Once written consent is received, patients are assigned and identified by a numeric code, which only select team members can decode.

Information collected for the study includes questionnaire responses, results from blood tests, and data from medical charts, including the Dossier de Santé Québec (DSQ), as well as notes from the lactation consultants recorded in the BSEI Activity Logs. A REDCap (https://projectredcap.org/software/) database has been designed and created for this project and is being used to capture the clinical case report forms, project questionnaires, and laboratory data. This facilitates tracking, statistics, and research into all aspects of the condition, as well as patient selection for potential clinical trials. Database information is confidential; REDCap is a secure, Health Insurance Portability and Accountability Act (HIPAA) compliant application used widely throughout North America for clinical studies and trials.

Sphygmo™ is a HIPAA and PIPEDA compliant platform. All information is encrypted on the app and in the server. The data is housed in a server located in Ontario with a company that specializes in medical data storage (Server Cloud Canada). Access to the user data is limited to what is necessary for maintenance and management of the platform. Otherwise, the only individuals who have access to study data are researchers with their individual credentials and accounts. Participants are provided a specific email address to upload BP values onto the Sphygmo™ application and do not use their personal email address. We believe this is a double safeguard to guarantee participants’ confidentiality.

Biomarker samples collected are not identified by name, but rather by the patient's assigned identification number. The samples will be stored at the Research Institute of the McGill University Health Center laboratory for 25 years after the end of the research project, after which they will be destroyed, unless participants consent for the use of their samples in future research studies.

### Knowledge translation

Our knowledge translation goals are to increase awareness of the importance of breastfeeding in vulnerable groups, and to promote integration of breastfeeding support as part of a global vascular risk reduction strategy in postpartum individuals. To attain these goals, we will reach out to members of the general public, by disseminating our findings to organizations dedicated to women’s health. Our results will also be presented to clinicians involved in perinatal care and policy makers.

## Discussion

HDP remains a prevalent pregnancy complication with long-term health sequelae for parent and child. While exclusive breastfeeding is recommended for at least 6 months with continued breastfeeding for up to 2 years for infant growth and immunologic health, there is a paucity of prospective high-quality data assessing the optimal duration for maternal health and child neurodevelopment. Although robust evidence for the maternal cardiometabolic benefits of breastfeeding is rapidly growing, there is a paucity of information on the impact of lactation on cardiovascular outcomes among individuals with HDP. Such information would not only contribute scientific understanding but would help to promote breastfeeding among individuals who may need it most, as a cost-effective postpartum intervention to lower maternal cardiometabolic risk. The sheMATTERS study has the potential to confirm such findings prospectively, and to further assess whether breastfeeding can help to reverse subclinical vascular impairment, which has never been systematically assessed.

Our goal is for the sheMATTERS study to contribute to improving postpartum cardiovascular health in new parents with hypertensive disorders of pregnancy. If our trial shows efficacy of the intervention, we will seek support for widespread implementation of supportive interventions for high-risk parents from public health agencies. Longer-term, our hope is that the sheMATTERS registry will help to identify personalized predictions of cardiovascular diseases after pregnancy and provide enhanced phenotyping of HDP in the postpartum period.

## Supplementary Information

**Additional file 1.** The MCQ captures socio-demographic and health information on participating families.

**Additional file 2.** At baseline, the Maternal CMF captures obstetrical information related to previous and the current pregnancy, as well as overall health status. At 6-weeks and 3-months the CMF is used to document the participant’s blood pressure. At 6- and 12-months the CMF is documents clinical measures (weight, blood pressure, hip and waist circumference) and blood and urine analyses.

**Additional file 3.** At baseline, the Infant CMF captures the infant’s health at the time of delivery. At subsequent time points, it captures the infant’s health and growth.

**Additional file 4: Appendix 1.** Trial Registration Data Set.

**Additional file 5: Appendix 2.** sheMATTERS Informed Consent Form.

**Additional file 6: Appendix 3.** Management of Biological Specimens.

## Data Availability

Aggregate de-identified data derived from SheMATTERS will be published in a peer-review journal in tabular form, with raw datasets available in spreadsheets as supplementary files.

## Abbreviations

HDP: Hypertensive disorder of pregnancy
BP: Blood pressure
ICU: Intensive care unit
BSE: Breastfeeding self-efficacy
BSES-SF: Breastfeeding self-efficacy scale- short form
RCT: Randomized control trial
BSEI: Breastfeeding self-efficacy intervention
CVD: Cardiovascular disease
NICU: Neonatal intensive care unit
HDL: High-density lipoprotein
LDL: Low-density lipoprotein
PBMC: Peripheral blood mononuclear cells
EBF: Exclusive breastfeeding
SBP: Systolic blood pressure
DBP: Diastolic blood pressure
AE: Adverse event
eCRF: Electronic case report form
SAE: Severe adverse event
REB: Research ethics board
