# Supplementary material for: Study protocol for the sheMATTERS study (iMproving cArdiovascular healTh in new moThERS): a randomized behavioral trial assessing the effect of a self-efficacy enhancing breastfeeding intervention on postpartum blood pressure and breastfeeding continuation in women with hypertensive disorders of pregnancy
Source: BMC Pregnancy Childbirth. 2023 Jan 26;23:68. doi: 10.1186/s12884-022-05325-3 (PMC9878496; doi:10.1186/s12884-022-05325-3)
Supplement: Supplementary file 1 — Additional file 1. The MCQ captures socio-demographic and health information on participating families. [file 12884_2022_5325_MOESM1_ESM.docx]

| **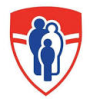** | i**M**proving c**A**rdiovascular heal**T**h in new mo**T**h**ERS**: “**she MATTERS**” | | | **INVESTIGATOR:**  Dr. Natalie Dayan  **CO-INVESTIGATOR:**  Dr. Sonia Semenic |
| --- | --- | --- | --- | --- |
|  | **PATIENT INITIALS:**  **________________________** First Middle Last | **STUDY ID #:**  **_______________________________** | **STUDY SITE:**  **________________________** | **PROTOCOL #:**    MP-37-2021-7201 |

# **MATERNAL CHARACTERISTICS QUESTIONNAIRE (MCQ) - Baseline**

Please complete the survey below which collects general information about you, your pregnancy and your health. 󠇃

Thank you!

**Date:** dd-mm-yyyy

**SECTION A: PREGNANCY INFORMATION**

__________________________________

A1. Who provided your care during your pregnancy? (Please select all that apply)

󠇃 Midwife

󠇃 Family physician

󠇃 Obstetrician

󠇃 Other

If other, please specify: (notes)

A2. Did you experience infertility prior to this pregnancy? (Note: infertility is defined as the inability to conceive after 12 months of unprotected intercourse)

󠇃 Yes

󠇃 No

󠇃 Not sure

**If NO or NOT SURE, go to A4**

A3. Which of the following was the cause of your infertility, to your knowledge? (Please select all that apply)

󠇃 Endometriosis

󠇃 Polycystic ovarian syndrome (PCOS)

󠇃 Blocked uterine tubes

󠇃 Uterine issue

󠇃 Hormonal issue

󠇃 Male factor

󠇃 Cause not known

󠇃 Other

If other, please specify: (notes)

A4. For how long were you trying to conceive before you got pregnant? (only one answer)

󠇃 Less than 6 months of unprotected intercourse

󠇃 6-12 months of unprotected intercourse

󠇃 More than 12 months of unprotected intercourse

󠇃 This pregnancy was not planned

󠇃 Not sure

A5. Did you use any infertility treatments (e.g., artificial insemination, in-vitro fertilization) or ovulation medications (e.g., clomiphene citrate, letrozole) to help you get pregnant?

󠇃 Yes

󠇃 No

󠇃 Not sure

**If NO or Not sure, go to A7**

A6. Please specify type of infertility treatment used for this pregnancy: (only one answer)

󠇃 Artificial insemination

󠇃 Ovulation medication only (e.g., clomiphene citrate, letrozole, metformin)

󠇃 Ovulation medication with artificial insemination

󠇃 Natural cycle artificial insemination

󠇃 In-vitro fertilization after hormonal stimulation

󠇃 In-vitro fertilization without hormonal stimulation

󠇃 Unsure

󠇃 Other

If other, please specify: (notes)

A7. Are there any medications that you took during this most recent pregnancy?

󠇃 Yes

󠇃 No

**If NO, end of section A**

A8. Please list all medications that you took during pregnancy

A8.1 Antithrombotic therapy

󠇃 Yes

󠇃 No

If yes,

A8.1a Please indicate the medication (drop down)

󠇃 ASA

󠇃 Dalteparin

󠇃 Enoxaparin

󠇃 Tinzaparin

󠇃 Fondaparinux

󠇃 Heparin

󠇃 Warfarin

󠇃 Other (note)

A81.b Please indicate the dose (drop down)

󠇃 (text) mcg

󠇃 (text) mg

󠇃 (text) g

󠇃 (text) ml

A8.1c Please indicate how do you take the medication (dropdown)

󠇃 Oral (by mouth)

󠇃 Subcutaneous (injection under the skin)

󠇃 Intravenous (injection in your vein)

󠇃 Intramuscular (in your muscles the way you receive the flu shot)

A8.1d Please indicate how often do you take the medication (dropdown)

󠇃 Once daily

󠇃 Twice daily

󠇃 Three times daily

󠇃 Four times daily

󠇃 Five times daily

󠇃 By hour (dropdown) 󠇃 󠇃

󠇃 Every 1h

󠇃 Every 2h

󠇃 Every 3h

󠇃 Every 4h

󠇃 Every 6h

󠇃 Every 8h

󠇃 Every 12h

󠇃 regularly

or 󠇃

󠇃 as needed

A8.1e Are you still taken the medication?

󠇃 Yes

󠇃 No

Date started (if known) (text)

**If marked No**

Date stopped (if known) (text)

A8.2 Antihypertensive therapy

󠇃 Yes

󠇃 No

If yes,

A8.2a Please indicate the medication (drop down)

󠇃 labetalol

󠇃 nifedipine XL

󠇃 nifedipine rapid acting

󠇃 hydralazine

󠇃 methyldopa

󠇃 amlodipine

󠇃 metoprolol

󠇃 propranolol

󠇃 bisoprolol

󠇃 furosemide

󠇃 hydrochlorothiazide

󠇃 enalapril

󠇃 ramipril

󠇃 other (note)

A8.2b Please indicate the dose (drop down)

󠇃 (text) mcg

󠇃 (text) mg

󠇃 (text) g

󠇃 (text) ml

A8.2c Please indicate how do you take the medication (dropdown)

󠇃 Oral (by mouth)

󠇃 Subcutaneous (injection under the skin)

󠇃 Intravenous (injection in your vein)

󠇃 Intramuscular (in your muscles the way you receive the flu shot)

A8.2d Please indicate how often do you take the medication (dropdown)

󠇃 Once daily

󠇃 Twice daily

󠇃 Three times daily

󠇃 Four times daily

󠇃 Five times daily

󠇃 By hour (dropdown) 󠇃 󠇃

󠇃 Every 1h

󠇃 Every 2h

󠇃 Every 3h

󠇃 Every 4h

󠇃 Every 6h

󠇃 Every 8h

󠇃 Every 12h

󠇃 regularly

or 󠇃

󠇃 as needed

A8.2e Are you still taken the medication?

󠇃 Yes

󠇃 No

Date started (if known) (text)

**If No**

Date stopped (if known) (text)

A8.3 Did you take other medications during this last pregnancy?

󠇃 Yes

󠇃 No

If yes, please complete the following information for each of the medications you are taking:

**If Yes, go to A8.3a**

**If No, end of section A**

A8.3a Please indicate the name of the medication (Text)

A8.3b Please indicate the dose (drop down)

󠇃 (text) mcg

󠇃 (text) mg

󠇃 (text) g

󠇃 (text) ml

A8.3c Please indicate how do you take the medication (dropdown)

󠇃 Oral (by mouth)

󠇃 Subcutaneous (injection under the skin)

󠇃 Intravenous (injection in your vein)

󠇃 Intramuscular (in your muscles the way you receive the flu shot)

A8.3d Please indicate how often do you take the medication (dropdown)

󠇃 Once daily

󠇃 Twice daily

󠇃 Three times daily

󠇃 Four times daily

󠇃 Five times daily

󠇃 By hour (dropdown) 󠇃 󠇃

󠇃 Every 1h

󠇃 Every 2h

󠇃 Every 3h

󠇃 Every 4h

󠇃 Every 6h

󠇃 Every 8h

󠇃 Every 12h

󠇃 regularly

or 󠇃

󠇃 as needed

A8.3e Are you still taken the medication?

󠇃 Yes

󠇃 No

Date started (if known) (text)

**If marked No**

Date stopped (if known) (text)

A8.4a Other medications?

󠇃 Yes

󠇃 No

**If yes**

A8.4a Please indicate the name of the medication (Text)

A8.4b Please indicate the dose (drop down)

󠇃 (text) mcg

󠇃 (text) mg

󠇃 (text) g

󠇃 (text) ml

A8.4c Please indicate how do you take the medication (dropdown)

󠇃 Oral (by mouth)

󠇃 Subcutaneous (injection under the skin)

󠇃 Intravenous (injection in your vein)

󠇃 Intramuscular (in your muscles the way you receive the flu shot)

A8.4d Please indicate how often do you take the medication (dropdown)

󠇃 Once daily

󠇃 Twice daily

󠇃 Three times daily

󠇃 Four times daily

󠇃 Five times daily

󠇃 By hour (dropdown) 󠇃 󠇃

󠇃 Every 1h

󠇃 Every 2h

󠇃 Every 3h

󠇃 Every 4h

󠇃 Every 6h

󠇃 Every 8h

󠇃 Every 12h

󠇃 regularly

or 󠇃

󠇃 as needed

A8.4e Are you still taken the medication?

󠇃 Yes

󠇃 No

Date started (if known) (text)

**If marked No**

Date stopped (if known) (text)

**SECTION B: INFORMATION ABOUT NEWBORN CONTACT AFTER DELIVERY**

This section asks about your experience with your baby right after childbirth.

B1. Was your baby placed in skin-to-skin contact with you within 5 minutes of delivery?

󠇃 Yes

󠇃 No

󠇃 Not sure

**If answer is YES or Not sure, go to B3**

B2. Why was your baby not placed skin-to-skin within 5 minutes of delivery?
󠇃 Baby required help with breathing

󠇃 Baby was taken to the special nursery or Neonatal Intensive Care Unit (NICU)

󠇃 Other

If other, please specify: (notes)

B3. Were you with your baby, without separation from you, for at least 1 hour after birth?

󠇃 Yes

󠇃 No

󠇃 Not sure

**If answer is YES or Not sure, go to B5**

B4. Why were you separated from your baby for the first hour of life?

󠇃 Baby required help with breathing

󠇃 Baby was taken to the special nursery or Neonatal Intensive Care Unit (NICU)

󠇃 Other

If other, please specify: (notes)

B5. Did you initiate breastfeeding within 1 hour of delivery?

󠇃 Yes

󠇃 No

󠇃 Not sure

B6. Was your baby admitted to the special care nursery or Neonatal Intensive Care Unit (NICU)?

󠇃 Yes

󠇃 No

󠇃 Not sure

**If No or not sure, End of section B**

B7: Please specify the reason for NICU admission: (notes)

**SECTION C: KNOWLEDGE ABOUT INFANT FEEDING**

The following section asks about the information you have received about breastfeeding, and any breastfeeding decisions you have made.

C1. During this most recent pregnancy, were you informed about the benefits of breastfeeding? 󠇃 󠇃 Yes

󠇃 No

󠇃 Not sure

C2. Are you planning on breastfeeding your baby?
󠇃 Yes

󠇃 No

**IF NO, End of section C**

C3. When did you decide to breastfeed your baby? (only one answer)

󠇃 Before becoming pregnant

󠇃 During the first trimester (up to 12 weeks)

󠇃 During the second trimester (12-28 weeks)

󠇃 During the last trimester (29 weeks to birth of baby)

󠇃 After birth of baby

󠇃 Not sure

C4. How long do you plan to breastfeed your baby?

󠇃 ____ months (text)

󠇃 For as long as possible

󠇃 Do not know/Not sure

C5. How long do you plan to EXCLUSIVELY breastfeed your baby?

󠇃 ____ months (text)

󠇃 For as long as possible

󠇃 Do not know/Not sure

C6. Did you breastfeed a previous child?

󠇃 Yes

󠇃 No

If yes, for how long? (text)

**SECTION D: GENERAL HEALTH**

The following section ask questions about your general health, including whether or not you have any health conditions that were present even before pregnancy, or that were diagnosed during pregnancy. This may include minor conditions (hypothyroidism managed with medication, minor anxiety) or more serious conditions (a heart condition).

D1. Has a doctor told you that you have high blood pressure?

󠇃 Yes

󠇃 No

󠇃 Not sure

D2. Have you been diagnosed with chronic hypertension?

󠇃 Yes

󠇃 No

󠇃 Not sure

D3. Have you been diagnosed with any other health condition, **not** related to blood pressure?

󠇃 Yes

󠇃 No

󠇃 Unsure

**If NO, go to D5**

D4. Please select **any** health conditions that you have been diagnosed with from the options below. You can select more than one condition that applies to you (please check all that apply)

󠇃 Chronic kidney disease

󠇃 Diabetes mellitus not requiring insulin

󠇃 Diabetes mellitus requiring insulin

󠇃 Heart arrhythmia

󠇃 Coronary artery disease

󠇃 Previous heart attack

󠇃 Other heart condition (specify) (text)

󠇃 Previous stroke

󠇃 Migraine

󠇃 Asthma

󠇃 Anxiety

󠇃 Depression

󠇃 Sleep Apnea

󠇃 Hypothyroidism

󠇃 Hyperthyroidism

󠇃 Liver cirrhosis

󠇃 Rheumatoid Arthritis

󠇃 Systemic Lupus Erythematosus

󠇃 Other Autoimmune condition (specify) (text)

󠇃 Cancer (specify) (text)

󠇃 Anemia

󠇃 Blood clot in the veins (pulmonary embolism or deep vein thrombosis)

󠇃 Human Immunodeficiency Virus (HIV)

󠇃 Hepatitis B infection

󠇃 Hepatitis C infection

If you have a health condition that is not listed, please type the health condition(s): (text)

D5. Do you currently take any prescription medications (including aspirin, asthma pump)?

󠇃 Yes

󠇃 No

**If NO, go to D6**

If yes, please complete the following information for each of the medications you are taking:

D5.1a Please indicate the name of the medication (Text)

D5.1b Please indicate the dose (drop down)

󠇃 (text) mcg

󠇃 (text) mg

󠇃 (text) g

󠇃 (text) ml

D5.1c Please indicate how do you take the medication (dropdown)

󠇃 Oral (by mouth)

󠇃 Subcutaneous (injection under the skin)

󠇃 Intravenous (injection in your vein)

󠇃 Intramuscular (in your muscles the way you receive the flu shot)

D5.1d Please indicate how often do you take the medication (dropdown)

󠇃 Once daily

󠇃 Twice daily

󠇃 Three times daily

󠇃 Four times daily

󠇃 Five times daily

󠇃 By hour (dropdown) 󠇃 󠇃

󠇃 Every 1h

󠇃 Every 2h

󠇃 Every 3h

󠇃 Every 4h

󠇃 Every 6h

󠇃 Every 8h

󠇃 Every 12h

󠇃 regularly

or 󠇃

󠇃 as needed

D5.1e Are you still taken the medication?

󠇃 Yes

󠇃 No

Date started (if known) (text)

**If marked No**

Date stopped (if known) (text)

D5.2 Other medications?

󠇃 Yes

󠇃 No

**If yes,**

D5.2a Please indicate the name of the medication (Text)

D5.2b Please indicate the dose (drop down)

󠇃 (text) mcg

󠇃 (text) mg

󠇃 (text) g

󠇃 (text) ml

D5.2c Please indicate how do you take the medication (dropdown)

󠇃 Oral (by mouth)

󠇃 Subcutaneous (injection under the skin)

󠇃 Intravenous (injection in your vein)

󠇃 Intramuscular (in your muscles the way you receive the flu shot)

D5.2d Please indicate how often do you take the medication (dropdown)

󠇃 Once daily

󠇃 Twice daily

󠇃 Three times daily

󠇃 Four times daily

󠇃 Five times daily

󠇃 By hour (dropdown) 󠇃 󠇃

󠇃 Every 1h

󠇃 Every 2h

󠇃 Every 3h

󠇃 Every 4h

󠇃 Every 6h

󠇃 Every 8h

󠇃 Every 12h

󠇃 regularly

or 󠇃

󠇃 as needed

D5.2e Are you still taken the medication?

󠇃 Yes

󠇃 No

Date started (if known) (text)

**If marked No**

Date stopped (if known) (text)

D6. Are you currently taking over the counter or herbal medication?

󠇃 Yes

󠇃 No

**If NO, go to D7**

**If Yes, go to D6.1a**

D6.1a Please indicate the name of the medication (Text)

D6.1b Please indicate the dose (drop down)

󠇃 (text) mcg

󠇃 (text) mg

󠇃 (text) g

󠇃 (text) ml

D6.1c Please indicate how do you take the medication (dropdown)

󠇃 Oral (by mouth)

󠇃 Subcutaneous (injection under the skin)

󠇃 Intravenous (injection in your vein)

󠇃 Intramuscular (in your muscles the way you receive the flu shot)

D6.1d Please indicate how often do you take the medication (dropdown)

󠇃 Once daily

󠇃 Twice daily

󠇃 Three times daily

󠇃 Four times daily

󠇃 Five times daily

󠇃 By hour (dropdown) 󠇃 󠇃

󠇃 Every 1h

󠇃 Every 2h

󠇃 Every 3h

󠇃 Every 4h

󠇃 Every 6h

󠇃 Every 8h

󠇃 Every 12h

󠇃 regularly

or 󠇃

󠇃 as needed

D6.1e Are you still taken the medication?

󠇃 Yes

󠇃 No

Date started (if known) (text)

**If marked No**

Date stopped (if known) (text)

D6.2 Other herbal medications?

󠇃 Yes

󠇃 No

D6.2a Please indicate the name of the medication (Text)

D6.2b Please indicate the dose (drop down)

󠇃 (text) mcg

󠇃 (text) mg

󠇃 (text) g

󠇃 (text) ml

D6.2c Please indicate how do you take the medication (dropdown)

󠇃 Oral (by mouth)

󠇃 Subcutaneous (injection under the skin)

󠇃 Intravenous (injection in your vein)

󠇃 Intramuscular (in your muscles the way you receive the flu shot)

D6.2d Please indicate how often do you take the medication (dropdown)

󠇃 Once daily

󠇃 Twice daily

󠇃 Three times daily

󠇃 Four times daily

󠇃 Five times daily

󠇃 By hour (dropdown) 󠇃 󠇃

󠇃 Every 1h

󠇃 Every 2h

󠇃 Every 3h

󠇃 Every 4h

󠇃 Every 6h

󠇃 Every 8h

󠇃 Every 12h

󠇃 regularly

or 󠇃

󠇃 as needed

D6.2e Are you still taken the medication?

󠇃 Yes

󠇃 No

Date started (if known) (text)

**If marked No**

Date stopped (if known) (text)

D7 Do you currently smoke?

󠇃 Yes

󠇃 No

**If NO, go to D9**

D8. Please specify number of cigarettes per day, on average:

󠇃 1–10

󠇃 11–20

󠇃 21–30

󠇃 31–40

󠇃 >40

D9. Have you ever smoked?

󠇃 Yes

󠇃 No

**If NO, go to D12**

D10. For how many years did you smoke? (number field, in years)

D11. Please specify number of cigarettes per day, on average

󠇃 1–10

󠇃 11–20

󠇃 21–30

󠇃 31–40

󠇃 >40

D12. Do you currently use cannabis in any form?

󠇃 Yes

󠇃 No

**If NO, go to D14**

D13. How often do you use cannabis? (only one answer)

󠇃 Every day

󠇃 More than once a week, but not every day

󠇃 Once a week

󠇃 Once a month

󠇃 Rarely or only on special occasions

D14. Do you use other recreational drugs?

󠇃 Yes

󠇃 No

**If Yes**, specify: (notes)

D15. Do you currently drink alcohol?

󠇃 Yes

󠇃 No

**If NO, end of section D**

D16. How often do you consume alcoholic beverages, on average?

󠇃 Every day

󠇃 Many times a week

󠇃 Once a week

󠇃 Only on weekends

󠇃 Rarely or only on special occasions

**SECTION E: COVID-19 STATUS**

E1. Were you ever tested for Covid-19?

󠇃 Yes

󠇃 No

**If NO, end of section E**

E2. When did you have your last test? dd mm yyyy

E3. Do you know the result of your most recent test?

󠇃 Positive

󠇃 Negative

󠇃 Not known yet

E4. What was the reason to have the test done?

- In contact with a person who tested positive
- Symptoms* compatible with COVID-19
- Test required by employer
- Travelled outside the country
- Other: specify (text)

*Symptoms include: fever, chills or history of fever, runny nose or nasal congestion, cough, sore throat, new onset headache, shortness of breath or respiratory difficulties, new onset diarrhea or vomiting, muscle and joint pain, loss of consciousness or sudden onset of weakness or fainting without reason.

**SECTION F: GENERAL INFORMATION**

The following are some general questions about yourself and your living situation.

F1. What is the highest level of education you completed?

󠇃 Elementary school

󠇃 Some high school

󠇃 High school

󠇃 Some CEGEP/community college/trade school

󠇃 Graduate of CEGEP/community college/trade school

󠇃 Some university (undergraduate degree)

󠇃 Completed university (undergraduate degree)

󠇃 Some graduate school

󠇃 Completed graduate degree

F2. Were you born in Canada?

󠇃 Yes

󠇃 No

󠇃 **If no**, where were you born? (city/town, country): (text)

󠇃 **If no**, how many years have you lived in Canada? _____ years (text)

F3. As you know, people living in Canada come from many different countries and cultures. Is there a particular ethnic or cultural group to which you belong? Select all that apply:

- North American
- Central or South American
- Caribbean
- European
- African
- West Central Asian or Middle Eastern
- East or Southeast Asian
- South Asian
- Aboriginal
- Do not wish to answer

F4. What is your current marital status?

󠇃 Married/common-law

󠇃 Single (never married)

󠇃 Divorced

󠇃 Separated

󠇃 Widowed

F5. Who is currently living with you? (please select all that apply)

󠇃 No one (living alone)

󠇃 spouse/partner

󠇃 children (other than your new baby)

󠇃 dependent adult family member

󠇃 non-dependent adult family member

󠇃 non-related adult (other than your partner)

󠇃 Other

**If other**, please specify: (notes)

F6. Did you work the year before you had your baby?

󠇃 Yes

󠇃 No

F7. What is your occupation? (notes)

F8. What is your annual household income before taxes?

(This figure will include income from any individuals living in your household and who contribute to the cost of you and your family's daily living. If you are currently on maternity leave, consider the income you had before your maternity leave.)

󠇃 $0 - $19,999

󠇃 $20,000 - $39,999

󠇃 $40,000 - $59,999

󠇃 $60,000 - $79,999

󠇃 $80,000 or more

󠇃 Do not wish to answer

Thank you for answering these questions.

# **MATERNAL CHARACTERISTICS QUESTIONNAIRE (MCQ) – 6 week, 3 month, 6 month and 12 month**

Please complete the survey below which collects general information about your health. 󠇃

Thank you!

**SECTION A: CURRENT PREGNANCY**

A1. Are you currently pregnant?

󠇃 Yes

󠇃 No

**If NO, go to section B**

**If YES to question A1, show question A2. End of questionnaire**

A2. How many weeks pregnant are you? (text)

**SECTION B: GENERAL HEALTH**

*At 6 weeks:* The following section ask questions about your general health **since you gave birth**. This may include minor conditions (hypothyroidism managed with medication, minor anxiety) or more serious conditions (a heart condition).

*At other follow up times:* The following section ask questions about your general health **since your last study visit**. This may include minor conditions (hypothyroidism managed with medication, minor anxiety) or more serious conditions (a heart condition).

B1. Has a doctor told you that you have high blood pressure?

󠇃 Yes

󠇃 No

󠇃 Not sure

B2. Have you been newly diagnosed with chronic hypertension?

󠇃 Yes

󠇃 No

󠇃 Not sure

B3. Have you been diagnosed with any other new health condition, **not** related to blood pressure?

󠇃 Yes

󠇃 No

󠇃 Unsure

**If NO, go to B5**

B4. Please select **any** new health conditions that you have been diagnosed with from the options below. You can select more than one condition that applies to you.

**(**please check all that apply)

󠇃 Chronic kidney disease

󠇃 Diabetes mellitus not requiring insulin

󠇃 Diabetes mellitus requiring insulin

󠇃 Heart arrhythmia

󠇃 Coronary artery disease

󠇃 Previous heart attack

󠇃 Other heart condition (specify) (text)

󠇃 Previous stroke

󠇃 Migraine

󠇃 Asthma

󠇃 Anxiety

󠇃 Depression

󠇃 Sleep Apnea

󠇃 Hypothyroidism

󠇃 Hyperthyroidism

󠇃 Liver cirrhosis

󠇃 Rheumatoid Arthritis

󠇃 Systemic Lupus Erythematosus

󠇃 Other Autoimmune condition (specify) (text)

󠇃 Cancer (specify) (text)

󠇃 Anemia

󠇃 Blood clot in the veins (pulmonary embolism or deep vein thrombosis)

󠇃 Human Immunodeficiency Virus (HIV)

󠇃 Hepatitis B infection

󠇃 Hepatitis C infection

If you have a new health condition that is not listed, please type the health condition(s): (text)

B5. Are you currently taking medications?

󠇃 Yes

󠇃 No

**If NO, end of questions about medications**

**If Yes, go to B5.1**

B5.1 Have your medications change since birth (if 3 months) / last visit (for all other time points)

󠇃 Yes

󠇃 No

**If NO, end of questions about medications**

**If Yes, go to B6**

B6. Do you currently take any prescription medications (including birth control medication, asthma pump)?

󠇃 Yes

󠇃 No

**If NO, go to B7**

B6.1a Please indicate the name of the medication (Text)

B6.1b Please indicate the dose (drop down)

󠇃 (text) mcg

󠇃 (text) mg

󠇃 (text) g

󠇃 (text) ml

B6.1c Please indicate how do you take the medication (dropdown)

󠇃 Oral (by mouth)

󠇃 Subcutaneous (injection under the skin)

󠇃 Intravenous (injection in your vein)

󠇃 Intramuscular (in your muscles the way you receive the flu shot)

B6.1d Please indicate how often do you take the medication (dropdown)

󠇃 Once daily

󠇃 Twice daily

󠇃 Three times daily

󠇃 Four times daily

󠇃 Five times daily

󠇃 By hour (dropdown) 󠇃 󠇃

󠇃 Every 1h

󠇃 Every 2h

󠇃 Every 3h

󠇃 Every 4h

󠇃 Every 6h

󠇃 Every 8h

󠇃 Every 12h

󠇃 regularly

or 󠇃

󠇃 as needed

B6.1e Are you still taken the medication?

󠇃 Yes

󠇃 No

Date started (if known) (text)

**If marked No**

Date stopped (if known) (text)

B6.2 Are you taking other medications?

󠇃 Yes

󠇃 No

**If yes,**

B6.2a Please indicate the name of the medication (Text)

B6.2b Please indicate the dose (drop down)

󠇃 (text) mcg

󠇃 (text) mg

󠇃 (text) g

󠇃 (text) ml

B6.2c Please indicate how do you take the medication (dropdown)

󠇃 Oral (by mouth)

󠇃 Subcutaneous (injection under the skin)

󠇃 Intravenous (injection in your vein)

󠇃 Intramuscular (in your muscles the way you receive the flu shot)

B6.2d Please indicate how often do you take the medication (dropdown)

󠇃 Once daily

󠇃 Twice daily

󠇃 Three times daily

󠇃 Four times daily

󠇃 Five times daily

󠇃 By hour (dropdown) 󠇃 󠇃

󠇃 Every 1h

󠇃 Every 2h

󠇃 Every 3h

󠇃 Every 4h

󠇃 Every 6h

󠇃 Every 8h

󠇃 Every 12h

󠇃 regularly

or 󠇃

󠇃 as needed

B6.2e Are you still taken the medication?

󠇃 Yes

󠇃 No

Date started (if known) (text)

**If marked No**

Date stopped (if known) (text)

B7. Are you currently taking over the counter or herbal medication?

󠇃 Yes

󠇃 No

**If NO, go to B8**

B7.1a Please indicate the name of the medication (Text)

B7.1b Please indicate the dose (drop down)

󠇃 (text) mcg

󠇃 (text) mg

󠇃 (text) g

󠇃 (text) ml

B7.1c Please indicate how do you take the medication (dropdown)

󠇃 Oral (by mouth)

󠇃 Subcutaneous (injection under the skin)

󠇃 Intravenous (injection in your vein)

󠇃 Intramuscular (in your muscles the way you receive the flu shot)

B7.1d Please indicate how often do you take the medication (dropdown)

󠇃 Once daily

󠇃 Twice daily

󠇃 Three times daily

󠇃 Four times daily

󠇃 Five times daily

󠇃 By hour (dropdown) 󠇃 󠇃

󠇃 Every 1h

󠇃 Every 2h

󠇃 Every 3h

󠇃 Every 4h

󠇃 Every 6h

󠇃 Every 8h

󠇃 Every 12h

󠇃 regularly

or 󠇃

󠇃 as needed

B6.1e Are you still taken the medication?

󠇃 Yes

󠇃 No

Date started (if known) (text)

**If marked No**

Date stopped (if known) (text)

B7.2 Other herbal medications?

󠇃 Yes

󠇃 No

**If yes,**

B7.2a Please indicate the name of the medication (Text)

B7.2b Please indicate the dose (drop down)

󠇃 (text) mcg

󠇃 (text) mg

󠇃 (text) g

󠇃 (text) ml

B7.2c Please indicate how do you take the medication (dropdown)

󠇃 Oral (by mouth)

󠇃 Subcutaneous (injection under the skin)

󠇃 Intravenous (injection in your vein)

󠇃 Intramuscular (in your muscles the way you receive the flu shot)

B7.2d Please indicate how often do you take the medication (dropdown)

󠇃 Once daily

󠇃 Twice daily

󠇃 Three times daily

󠇃 Four times daily

󠇃 Five times daily

󠇃 By hour (dropdown) 󠇃 󠇃

󠇃 Every 1h

󠇃 Every 2h

󠇃 Every 3h

󠇃 Every 4h

󠇃 Every 6h

󠇃 Every 8h

󠇃 Every 12h

󠇃 regularly

or 󠇃

󠇃 as needed

B7.2e Are you still taken the medication?

󠇃 Yes

󠇃 No

Date started (if known) (text)

**If marked No**

Date stopped (if known) (text)

B8. Have you been hospitalized for any reason?

󠇃 Yes

󠇃 No

**If NO, go to B13.**

B9. What was the date of hospitalization: Dd/mm/yyyy

B10. What was the reason for hospitalization? (notes)

B11. How long were you hospitalized for?

󠇃 Less than 1 week

󠇃 1 to 2 weeks

󠇃 More than 2 weeks

B12 Do you currently smoke?

󠇃 Yes

󠇃 No

**If NO, go to B15**

B13. Please specify number of cigarettes per day, on average:

󠇃 1–10

󠇃 11–20

󠇃 21–30

󠇃 31–40

󠇃 >40

B14. Do you currently use cannabis in any form?

󠇃 Yes

󠇃 No

**If NO, go to B16**

B15. How often do you use cannabis? **(only one answer)**

󠇃 Every day

󠇃 More than once a week, but not every day

󠇃 Once a week

󠇃 Once a month

󠇃 Rarely or only on special occasions

B16. Do you use other recreational drugs?

󠇃 Yes

󠇃 No

**If Yes**, specify: (notes)

B17. Do you currently drink alcohol?

󠇃 Yes

󠇃 No

**If NO, end of section B**

B18. How often do you consume alcoholic beverages, on average?

󠇃 Every day

󠇃 Many times a week

󠇃 Once a week

󠇃 Only on weekends

󠇃 Rarely or only on special occasions

**SECTION C: COVID-19 STATUS**

C1. Were you tested for Covid-19 since last visit?

󠇃 Yes

󠇃 No

**If NO, end of questionnaire**

C2. When did you have your test?: dd mm yyyy

C3. Do you know the result of your test?

󠇃 Positive

󠇃 Negative

󠇃 Not known yet

C4. What was the reason to have the test done?

- In contact with a person who tested positive
- Symptoms* compatible with COVID-19
- Test required by employer
- Travelled outside the country
- Other: specify (text)

*Symptoms include: fever, chills or history of fever, runny nose or nasal congestion, cough, sore throat, new onset headache, shortness of breath or respiratory difficulties, new onset diarrhea or vomiting, muscle and joint pain, loss of consciousness or sudden onset of weakness or fainting without reason.

Thank you for answering these questions.
