# Supplementary material for: Study protocol for the sheMATTERS study (iMproving cArdiovascular healTh in new moThERS): a randomized behavioral trial assessing the effect of a self-efficacy enhancing breastfeeding intervention on postpartum blood pressure and breastfeeding continuation in women with hypertensive disorders of pregnancy
Source: BMC Pregnancy Childbirth. 2023 Jan 26;23:68. doi: 10.1186/s12884-022-05325-3 (PMC9878496; doi:10.1186/s12884-022-05325-3)
Supplement: Supplementary file 2 — Additional file 2. At baseline, the Maternal CMF captures obstetrical information related to previous and the current pregnancy, as well as overall health status. At 6-weeks and 3-months the CMF is used to document the participant’s blood pressure. At 6- and 12-months the CMF is documents clinical measures (weight, blood pressure, hip and waist circumference) and blood and urine analyses. [file 12884_2022_5325_MOESM2_ESM.docx]

| **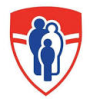** | i**M**proving c**A**rdiovascular heal**T**h in new mo**T**h**ERS**: “**she MATTERS**” | | | **INVESTIGATOR:**  Dr. Natalie Dayan  **CO-INVESTIGATOR:**  Dr. Sonia Semenic |
| --- | --- | --- | --- | --- |
|  | **PATIENT INITIALS:**  **________________________** First Middle Last | **STUDY ID #:**  **_______________________________** | **STUDY SITE:**  **________________________** | **PROTOCOL #:**    MP-37-2021-7201 |

**CLINICAL MEASURES FORM – MOTHER**

**Baseline**

**To be completed by research personnel via chart review**

**SECTION Aa: MENTAL HEALTH STATUS AND FOLLOW UP**

**Aa1**. EPDS and STAI done?

 Yes

 No

**Aa2**. Psychology referral made? (NA = when in normal range)

 Yes

 No

**SECTION A: GENERAL INFORMATION**

**A1**. Date of measurements recorded: dd/mm/yyyy

**A2**. Treating obstetrician, caregiver, or midwife:

Name: (text)

License: (text)

Address: (text)

Phone number: (text)

**A3**. Obstetrician attending delivery:

Name: (text)

License: (text)

**A4**. Mother’s DOB: dd/mm/yyyy

**A5.** Mother’s age : _____ years

**A5**. Infant’s DOB: dd/mm/yyyy

**A6.** Date of hospital admission (hospitalization for delivery): dd/mm/yyyy

**A7.** Date of hospital discharge (hospitalization for delivery): dd/mm/yyyy

**SECTION B. OBSTETRIC INFORMATION FOR CURRENT PREGNANCY**

**B1.** Timing of first antenatal weight recorded in chart:

 Weeks gestation: _____ (text)

 Not recorded

**B2.** First antenatal weight recorded:

 ___ ___ ___ kg

 ___ ___ ___ lb

 Not recorded

**B3**. Weight gain during pregnancy:

 ___ ___ ___ kg

 ___ ___ ___ lb

 Not recorded

**B4.** Timing of first recorded antenatal blood pressure:

 Weeks gestation: ______ (text)

 Not recorded

 Not available

**B5.** First recorded systolic blood pressure (mmHg): (text)

**B6.** First recorded diastolic blood pressure (mmHg): (text)

**B7.** Gestational age at diagnosis of hypertensive disorder of pregnancy:

___ weeks ___ days

**B8.** Gestational age at delivery:

___ weeks ___ days

**B9.** Gestational age calculated based on:

 Last Menstrual Period

 Ultrasound

 Unknown

**B10.** Type of hypertensive disorder of pregnancy (choose one of the following):

 Chronic hypertension without worsening in pregnancy

 Chronic hypertension with worsening in pregnancy

* Chronic hypertension **with superimposed** **preeclampsia**

 Gestational hypertension without preeclampsia

* Gestational hypertension **with superimposed preeclampsia**

* **Preeclampsia**

**If *marked, show B11. If not, go to B12**

**B11.** Adverse condition or severe complication (choose all that apply)

 Hemolysis elevated liver enzymes low platelets (HELLP) syndrome

 Eclampsia

 Stroke

 Intracranial hemorrhage

 Coma

 Encephalopathy or delirium

 Myocardial infarction

 Pulmonary edema or heart failure

 Arrhythmia

 Kidney failure

 Dialysis

 Disseminated intravascular coagulation

 Hepatic hematoma

 Fetal growth restriction (<5^th^ percentile) on ultrasound

 Other (specify): (text)

**If Fetal Growth restriction is marked:**

Date: dd/mm/yyyy

**B12.** Other complication in study pregnancy or at delivery (select all that apply):

 Gestational diabetes

 Preterm birth (premature, less than 37 weeks)

 Low birth weight (less than 2500 grams)

 Postpartum hemorrhage

 Maternal ICU admission

 Transfusion

 Sepsis/severe infection

 Venous thromboembolism (deep venous thrombosis, pulmonary embolism, cerebral vein thrombosis, pelvic vein thrombosis)

 Stroke

 Heart failure

 Myocardial infarction

 Surgical complication

 Other: Specify (text)

 None of the above

**B13.** Type of labor

 Induced

 Spontaneous

**B14.** Length of labor : (HH:MM)

**B15.** Mode of delivery for study pregnancy (select one only):

 Unassisted vaginal birth

 Vaginal birth with vacuum extraction or forceps

 Elective Caesarean-section (C-section) (booked or scheduled in advance)

 C-section with labour (emergency or unplanned)

 Unclear

**If C-section with labour,** Specify reason for emergency delivery (please select all that apply):

 Fetal well-being

 Maternal well-being

 Blood pressure too high

 Other Please specify: (text)

 Unclear

**SECTION C. PREVIOUS PREGNANCIES**

**C1.** Number of previous pregnancies (gravidity): (text) [range 0 to 20]

**C2.** Number of previous deliveries > 20 weeks (parity): (text) [range 0 to 20]

**C3.** Number of previous miscarriages < 10 weeks: (text) [range 0 to 20]

**C4.** Number of previous fetal losses between 10 weeks and 19 weeks+6 days: (text) [range 0 to 20]

**C5.** Number of prior abortions: (text) [range 0 to 20]

**C6.** Number of living biological children: (text) [range 0 to 20]

**C7.** Information about previous pregnancies  **Skip If C2=0)**

**Pregnancy 1, 2, 3 …20:**

- Gestational age at delivery (weeks): (text)
- Date of delivery: dd/mm/yyyy
- Type of labor

󠇃Induced/

󠇃Spontaneous

- Type of delivery

 Vaginal birth

 Vaginal birth with vacuum extraction or forceps

 Elective caesarean-section (booked or scheduled in advance)

 Caesarean-section with labour (emergency or unplanned)

**If emergency C-section,** specify reason for emergency delivery (please select all that apply):

 Fetal well-being

 Maternal well-being

 Blood pressure too high

 Other please specify: (text)

 Unclear

- Delivery outcome

 Live birth

 Stillbirth

- Neonatal complication (select any that apply):

 Neonatal ICU admission

 Acute Respiratory Distress Syndrome

 Intracranial hemorrhage

 Retinopathy of prematurity

 Necrotizing enterocolitis

 Meconium aspiration

 Hypoglycemia

 Jaundice

 Neonatal death < 28 days

 Congenital anomaly

 Other (specify): (text)

- Birthweight (g) (text) [range 400 – 5000]
- Infant sex

 M

 F

 Other: (text)

- Maternal complications in pregnancy (select any that apply)

 Chronic hypertension without worsening in pregnancy

 Chronic hypertension with worsening in pregnancy

 Chronic hypertension with superimposed preeclampsia

 Gestational hypertension without preeclampsia

 Gestational hypertension with superimposed preeclampsia

 Preeclampsia

 Hemolysis elevated liver enzymes low platelets (HELLP) syndrome

 Eclampsia

 Stroke

 Intracranial hemorrhage

 Coma

 Encephalopathy or delirium

 Myocardial infarction

 Pulmonary edema or heart failure

 Arrhythmia

 Kidney failure

 Dialysis

 Disseminated intravascular coagulation

 Hepatic hematoma

 Gestational diabetes

 Preterm birth (premature, less than 37 weeks)

 Postpartum hemorrhage

 Maternal ICU admission

 Transfusion

 Sepsis/severe infection

 Venous thromboembolism (deep venous thrombosis, pulmonary embolism, cerebral vein thrombosis, pelvic vein thrombosis)

 Surgical complication

 Other Specify: (text)

**SECTION D: BASELINE ANTHROPOMETRIC MEASUREMENTS**

**D1**. Current weight:

 ___ kg

 ___ lb

 Not recorded

**D2**. Current height:

 ____feet ___ inches

 ___ cm

 Not recorded/not measured

**D3.** Source of data for weight

 Measured

 Self-reported

 Chart

**D4.** Source of data for height

 Measured

 Self-reported

 Chart

**SECTION E: VITAL SIGNS**

Acceptable range for systolic blood pressure 85 – 240 mmHg

Acceptable range for diastolic blood pressure 50 – 140 mmHg

Acceptable range for pulse 35 – 200 bpm

Acceptable range for oxygen saturation 60 – 100%

**E1.** Mean values from SunTech machine:

 Systolic blood pressure: ___ ___ mmHg

 Diastolic blood pressure: ___ ___ mmHg

 Pulse: _____ beats per minute

 Side:

 Position:

**E2**. Date and time values taken:

 ___ / ___ / _____ @ ___h ___

dd mm yyyy hh mm

 Not recorded

**E3.** Measurements from A&D Bluetooth-enabled machine:

 Systolic: (text) mmHg

 Diastolic: (text) mmHg

 Side:

 Not recorded

 Pulse: (text) beats per minute

**E4**. Date and time values taken:

 ___ / ___ / ___ @ ___h ___

dd mm yy hh mm

 Not recorded

**E5.** Most recent vital signs from medical chart at time of enrollment in study

 Systolic: ___ ___ mmHg

 Diastolic: ___ ___ mmHg

 Pulse: _____ beats per minute

 Temperature:________ degrees Celcius

 Oxygen saturation: ________%

 Oxygen supplementation nasal cannula: ______liters per minute

 Oxygen supplementation face mask: ________liters per minute

**E6**. Date and time recent values taken:

 ___ ___ ___ / @ ___h ___

dd mm yy hh mm

 Not recorded

**E7. Highest** blood pressure value from medical chart at time of enrollment in study

 Systolic: ___ ___ ___ mmHg

 Diastolic: ___ ___ ___ mmHg

 Pulse at the time of highest BP value: _____ beats per minute

**E8**. Date and time highest values taken:

 ___ ___ ___ / @ ___h ___

dd mm yy hh mm

 Not recorded

**E9.** Blood pressure value from medical chart at time of diagnosis of hypertensive disorder of pregnancy

 Systolic: (text) mmHg

 Diastolic: (text) mmHg

 Pulse at the time of diagnosis: (text) beats per minute

**E10**. Date and time blood pressure values taken at time of diagnosis:

 ___ ___ ___ / @ ___h ___

dd mm yy hh mm

 Not recorded

**E11.** During this hospitalization, did the patient have any of the following abnormal vital signs? (mark all that apply)

 Severe systolic hypertension (systolic blood pressure > 160 mmHg)

 Severe diastolic hypertension (diastolic blood pressure > 110 mmHg)

 Severe systolic hypotension (systolic blood pressure < 90 mmHg)

 Severe diastolic hypotension (diastolic blood pressure < 60mmHg)

 Severe tachycardia (pulse > 120 beats per minute)

 Severe bradycardia (pulse < 50 beats per minute)

 Fever (Temperature > 38.5 degrees Celcius)

 Hypothermia (Temperature < 35.0 degrees Celcius)

 Hypoxia (oxygen saturation < 90% breathing ambient air)

 Oxygen supplementation (add litres per minute)

**For each abnormal vital sign in E11 that is ticked off,**

**E12.** Please provide Date and Time

 ___ ___ ___ / @ ___h ___

dd mm yy hh mm

 Not recorded

**E13**. Vital sign notes**:** (notes)

**SECTION F: MEDICATION USE**

**F1.** Did the participant receive acetyl-salicylic acid (aspirin, ASA) during pregnancy?

 Yes

 No

**If NO go to F3.**

**F2.** Please answer the following about ASA use:

Dose

󠇃 81 mg

󠇃 162 mg

󠇃 Unknown

󠇃 Other

Frequency

󠇃 Daily, any time

󠇃 Daily, at night

󠇃 BID

Gestational age started (weeks): (text)

Gestational age stopped (weeks): (text)

**F3.** Did the participant receive Magnesium Sulphate (MgSO4) infusion for severe preeclampsia or eclampsia during this hospitalization?

 Yes

 No

**If NO go to F5**

**F4.** Date and Time of MgSO4 initiation?

 ___ ___ ___ / @ ___h ___

dd mm yy hh mm

 Duration of MgSO4 infusion (number field, hours)

**F5.** Did the patient receive anti-hypertensive medication in hospital?

 Yes

 No

**If NO then F7**

**F6.** Please indicate anti-hypertensive medication used in hospital

- Labelatol
- Methyldopa
- Nifedipine XL
- Nifedipine rapid acting
- Hydralazine
- Enalapril
- Other: specify (text)

**For each medication selected** Please indicate:

- Dose (mg) : (text)
- Frequency: (text)
- Route (po/IV/subcutaneous/sublingual): (text)
- Date prescribed: dd/mm/yyyy

**F7.** Please indicate any medications used in hospital **(Possibility to duplicate if multiple medication)**

- Medication: (text)
- Dose (mg) : (text)
- Frequency: (text)
- Route (po/IV/subcutaneous/sublingual): (text)
- Date prescribed: dd/mm/yyyy

**F8.** Please indicate any medications prescribed upon discharge from hospital **(Possibility to duplicate if multiple medication)**

- Medication: (text)
- Dose (mg) : (text)
- Frequency: (text)
- Route (po/IV/subcutaneous/sublingual): (text)
- Date prescribed: dd/mm/yyyy

**SECTION G: BLOOD AND URINE COMPOSITION**

**Please insert the most recent blood test values at the time of completion of the CMF (from participants’ medical chart).**

**Date of laboratory test:** dd/mm/yyyy

G1. Hemoglobin level (text) (g/L) dd/mm/yyyy

G2. Platelet level (text) (x 109/L)

G3. White blood cell count (text) (x 109/L)

G4. Bilirubin (text) (mg/dl)

G5. Alanine aminotransferase (ALT) (text) (IU/L)

G6. Aspartate aminotransferase (AST) (text) (IU/L)

G7. Alkaline phosphatase (ALP) (text) (IU/L)

G8. Creatinine

󠇃 (text) µmol/L or

󠇃 (text) mg/dL

G9. Serum electrolytes

󠇃 Sodium

󠇃 (text) mEq/L

󠇃 (text) mmol/L

󠇃 Potassium

󠇃󠇃 (text) mEq/L

󠇃 (text) mmol/L

󠇃 Chloride (text) mmol/L

󠇃 Bicarbonate 󠇃

󠇃 (text) mEq/L

󠇃 (text) mmol/L

G10. Total Calcium 󠇃

󠇃(text) mg/dL or

󠇃󠇃(text) mmol/L

G11. Albumin level

󠇃󠇃 (text) g/dL

󠇃 (text)g/L

G12. Magnesium level 󠇃 󠇃

󠇃 (text) mEq/L

󠇃 (text) mmol/L

G13. Uric acid 󠇃 󠇃(text) mg/dL

G14. Lactate dehydrogenase 󠇃(text) (LDH) U/L

G15. High-sensitivity troponin 󠇃(text) ng/mL

G16. Troponin-I 󠇃 (text) ng/mL

G17. Brain Natriuretic Peptide 󠇃 (text) (pg/ml)

G18. Urine protein: creatinine ratio: (text) mg/mmol

**SECTION H: BIOMARKERS**

H1: Was sample obtained?

Yes

 No

**If yes:**

- Serum:
- PMBC:

H2: Additional notes: (notes)

**CLINICAL MEASURES FORM – Mother**

**6 WEEK**

**SECTION A: VITAL SIGNS**

Acceptable range for systolic blood pressure 85 – 240 mmHg

Acceptable range for diastolic blood pressure 50 – 140 mmHg

Acceptable range for pulse 35 – 200 bpm

**A1.** Measurements from A&D Bluetooth-enabled machine:

 Systolic: (text) mmHg

 Diastolic: (text) mmHg

 Not recorded

 Pulse: (text) beats per minute

**A2**. Date and time values taken:

 ___ / ___ / ___ @ ___h ___

dd mm yy hh mm

 Not recorded

**CLINICAL MEASURES FORM – Mother**

**3 MONTH**

**SECTION Aa: MENTAL HEALTH STATUS AND FOLLOW UP**

**Aa1**. EPDS and STAI done?

 Yes

 No

**Aa2**. Psychology referral made? (NA = when in normal range)

 Yes

 No

**SECTION B: VITAL SIGNS**

Acceptable range for systolic blood pressure 85 – 240 mmHg

Acceptable range for diastolic blood pressure 50 – 140 mmHg

Acceptable range for pulse 35 – 200 bpm

**B1.** Measurements from A&D Bluetooth-enabled machine:

 Systolic: (text) mmHg

 Diastolic: (text) mmHg

 Not recorded

 Pulse: (text) beats per minute

**B2**. Date and time values taken:

 ___ / ___ / ___ @ ___h ___

dd mm yy hh mm

 Not recorded

**CLINICAL MEASURES FORM – Mother**

**6 MONTH**

**SECTION Aa: MENTAL HEALTH STATUS AND FOLLOW UP**

**Aa1**. EPDS and STAI done?

 Yes

 No

**Aa2**. Psychology referral made? (NA = when in normal range)

 Yes

 No

**SECTION A: ANTHROPOMETRIC MEASUREMENTS**

**A1**. Current weight:

 ___ kg

 ___ lb

 Not recorded

**A2.** Source of data for weight

 Measured

 Self-reported

 Chart

**A3**. Waist measurement

- ______ inches

**A4**. Hips measurement

- ______ inches

**SECTION B: VITAL SIGNS**

Acceptable range for systolic blood pressure 85 – 240 mmHg

Acceptable range for diastolic blood pressure 50 – 140 mmHg

Acceptable range for pulse 35 – 200 bpm

**B1.** Mean values from SunTech machine (if “on-site” visit)

 Systolic blood pressure: ___ ___ mmHg

 Diastolic blood pressure: ___ ___ mmHg

 Pulse: _____ beats per minute

**B2**. Date and time values taken:

 ___ / ___ / _____ @ ___h ___

dd mm yyyy hh mm

 Not recorded

**B3.** Mean values from A&D machine

 Systolic blood pressure: ___ ___ mmHg

 Diastolic blood pressure: ___ ___ mmHg

 Pulse: _____ beats per minute

**B4**. Date and time values taken:

 ___ / ___ / _____ @ ___h ___

dd mm yyyy hh mm

 Not recorded

**SECTION C: BLOOD AND URINE COMPOSITION**

**Date of laboratory test done at 6-month visit:** dd/mm/yyyy

Biochemistry

**C1**. Serum electrolytes:

- Sodium:
  - (text) mEq/L
  - (text) mmol/L
- Potassium:
  - (text) mEq/L
  - (text) mmol/L
- Chloride:
  - (text) mmol/L
- Total Calcium
  - (text) mg/dL or 󠇃
  - (text) mmol/L)

**C2.** Fasting Glucose (mmol/L)

**C3.** Random Glucose (mmol/L)

**C4.** Hemoglobin A1C (mmol/L)

**C5**. Creatinine

- (text) µmol/L or󠇃
- (text) mg/dL

**C6**. Lipid Profile

- Cholesterol (text) (mmol/L)
- Tryglicerides (text) (mmol/L)
- HDL(text) (mmol/L)
- LDL (text) (mmol/L)
- Non HDL C (mmol/L)
- Apolipoprotein B (g/L)

**C7**. Endocrinology- Thyroid Profile

- TSH (text) (mIU/L)
- T4 (if TSH is abnormal) (text) (μg/dL)

**C8.** Micro-albumin Urine Random Profile

- Protein (g/L) (text)
- Micro-albumin (mg/L) (text)
- Creatinine (mmol/L) (text)
- Micro-albumin/ Creatinine (mg/mmol) (text)
- Urine protein: creatinine ratio: (text)
- Micro-albumin/ Creatinine Ratio (g/mmol) (text)
- Protein/ Creatinine (g/g) (text)

**C9**. Urine Analysis

- Appearance: (text)
- Color: (text)
- Specific gravity: (text)
- Bilirubin: (text)
- Blood: (text)
- Glucose: (text)
- Ketones: (text)
- Leukocyte Esterasa: (text)
- Nitrite: (text)
- pH: (text)
- Protein: (text)
- Urobilinogen: (text)
- UA RBC Automated Microscopy: (text)
- UA WBC Automated Microscopy: (text)
- UA Hyaline Casts Automated Microscopy: (text)
- UA Bacteria Automated Microscopy: (text)
- UA Epithelial Cells Automated Microscopy: (text)

**C10**. Are more blood tests needed related to hypertension (determined by PI based on Blood Pressure values and other clinical characteristics)?

󠇃 Yes

󠇃 No

**If YES**

- aldosterone concentration (text) ng/dL
- renin concentration (text) ng/mL/hour
- cortisol concentration (text) μg/dL
- urine and/or plasma metanephrines (text) pg/ml
- antiphospholipid syndrome lupus anticoagulant (text) Units
- IgG and IgM anti-cardiolipin antibodies (text) Units
- beta-2 glycoprotein-1(text) Units
- Other (text)

**C11**: Additional notes: (notes)

**CLINICAL MEASURES FORM – Mother**

**12 MONTH**

**SECTION Aa: MENTAL HEALTH STATUS AND FOLLOW UP**

**Aa1**. EPDS and STAI done?

 Yes

 No

**Aa2**. Psychology referral made? (NA = when in normal range)

 Yes

 No

**SECTION A: ANTHROPOMETRIC MEASUREMENTS**

**A1**. Current weight:

 ___ kg

 ___ lb

 Not recorded

**A2.** Source of data for weight

 Measured

 Self-reported

 Chart

**A3**. Waist measurement

- ______ inches

**A4**. Hips measurement

- ______ inches

**SECTION B: VITAL SIGNS**

Acceptable range for systolic blood pressure 85 – 240 mmHg

Acceptable range for diastolic blood pressure 50 – 140 mmHg

Acceptable range for pulse 35 – 200 bpm

**B1.** Mean values from SunTech machine (if “on-site” visit)

 Systolic blood pressure: ___ ___ mmHg

 Diastolic blood pressure: ___ ___ mmHg

 Pulse: _____ beats per minute

**B2**. Date and time values taken:

 ___ / ___ / _____ @ ___h ___

dd mm yyyy hh mm

 Not recorded

**B3.** Mean values from A&D machine

 Systolic blood pressure: ___ ___ mmHg

 Diastolic blood pressure: ___ ___ mmHg

 Pulse: _____ beats per minute

**B4**. Date and time values taken:

 ___ / ___ / _____ @ ___h ___

dd mm yyyy hh mm

 Not recorded

**SECTION C: BLOOD AND URINE COMPOSITION**

**Date of laboratory test done at 6-month visit:** dd/mm/yyyy

Biochemistry

**C1**. Serum electrolytes:

- Sodium:
  - (text) mEq/L
  - (text) mmol/L
- Potassium:
  - (text) mEq/L
  - (text) mmol/L
- Chloride:
  - (text) mmol/L
- Total Calcium
  - (text) mg/dL or 󠇃
  - (text) mmol/L)

**C2.** Fasting Glucose (mmol/L)

**C3.** Random Glucose (mmol/L)

**C4.** Hemoglobin A1C (mmol/L)

**C5**. Creatinine

- (text) µmol/L or󠇃
- (text) mg/dL

**C6**. Lipid Profile

- Cholesterol (text) (mmol/L)
- Tryglicerides (text) (mmol/L)
- HDL(text) (mmol/L)
- LDL (text) (mmol/L)
- Non HDL C (mmol/L)
- Apolipoprotein B (g/L)

**C7**. Endocrinology- Thyroid Profile

- TSH (text) (mIU/L)
- T4 (if TSH is abnormal) (text) (μg/dL)

**C8.** Micro-albumin Urine Random Profile

- Protein (g/L) (text)
- Micro-albumin (mg/L) (text)
- Creatinine (mmol/L) (text)
- Micro-albumin/ Creatinine (mg/mmol) (text)
- Urine protein: creatinine ratio: (text)
- Micro-albumin/ Creatinine Ratio (g/mmol) (text)
- Protein/ Creatinine (g/g) (text)

**C9**. Urine Analysis

- Appearance: (text)
- Color: (text)
- Specific gravity: (text)
- Bilirubin: (text)
- Blood: (text)
- Glucose: (text)
- Ketones: (text)
- Leukocyte Esterasa: (text)
- Nitrite: (text)
- pH: (text)
- Protein: (text)
- Urobilinogen: (text)
- UA RBC Automated Microscopy: (text)
- UA WBC Automated Microscopy: (text)
- UA Hyaline Casts Automated Microscopy: (text)
- UA Bacteria Automated Microscopy: (text)
- UA Epithelial Cells Automated Microscopy: (text)

**C10**. Are more blood tests needed related to hypertension (determined by PI based on Blood Pressure values and other clinical characteristics)?

󠇃 Yes

󠇃 No

**If YES**

- aldosterone concentration (text) ng/dL
- renin concentration (text) ng/mL/hour
- cortisol concentration (text) μg/dL
- urine and/or plasma metanephrines (text) pg/ml
- antiphospholipid syndrome lupus anticoagulant (text) Units
- IgG and IgM anti-cardiolipin antibodies (text) Units
- beta-2 glycoprotein-1(text) Units
- Other (text)

**C11**: Additional notes: (notes)

**SECTION D: BIOMARKERS**

D1: Was sample obtained?

Yes

 No

**If yes:**

Serum:

PMBC:

D2: Additional notes: (notes)
