# Supplementary material for: Study protocol for the sheMATTERS study (iMproving cArdiovascular healTh in new moThERS): a randomized behavioral trial assessing the effect of a self-efficacy enhancing breastfeeding intervention on postpartum blood pressure and breastfeeding continuation in women with hypertensive disorders of pregnancy
Source: BMC Pregnancy Childbirth. 2023 Jan 26;23:68. doi: 10.1186/s12884-022-05325-3 (PMC9878496; doi:10.1186/s12884-022-05325-3)
Supplement: Supplementary file 3 — Additional file 3. At baseline, the Infant CMF captures the infant’s health at the time of delivery. At subsequent time points, it captures the infant’s health and growth. [file 12884_2022_5325_MOESM3_ESM.docx]

| **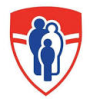** | i**M**proving c**A**rdiovascular heal**T**h in new  mo**T**h**ERS**: “**she MATTERS**” | | | **INVESTIGATOR:**  Dr. Natalie Dayan  **CO-INVESTIGATOR:**  Dr. Sonia Semenic |
| --- | --- | --- | --- | --- |
|  | **PATIENT INITIALS:**  **________________** | **STUDY ID #:**  **___________** | **STUDY SITE:**  **________________________** | **PROTOCOL #:**    MP-37-2021-7201 |

**Date**: dd-mm-yyyy

**CLINICAL MEASURES FORM – INFANT**

**BASELINE**

**1.** Sex of infant:

 Male

 Female

 Discrepancy in sexual differentiation / Unknown

 Not recorded

**2.** Gestational age at birth: ____Weeks (number field 22-44) ____Days (number field 0-6)

**3.** APGAR score:

 1 minute: (number field 0-10)

 5 minutes: (number field 0-10)

 10 minutes: (number field 0-10)

**4.** Preterm birth? (i.e., born at less than 37 weeks of estimated gestational age)

 Yes

 No

**5.** Birth weight of infant:

 _____ grams (number field 300-6000) OR

 ______pounds(lb) ____ ounces(oz) (number field)

**6.** Birthweight calculated for gestational age:

 ___ ___ ___ ___ g

**7.** Neonatal length: ___ ___ ___ cm (number field)

**8.** Neonatal head circumference: ___ ___ ___ cm (number field)

**9.** Cord blood pH: ________ (number field 6.70-7.50)

**10.** Cord pCO2: (text) mmHg

**11.** Cord Base Excess: (text) mmol/L

**12.** Cord Bicarbonate: (text) (mmol/L)

**13**. Placental pathology

 Yes

 No

**If YES:**

**14**. Placental pathology description

 Advanced villous maturation

 Vasculopathy

 Infarction

 Hypoperfusion

 Other: (text)

**15.** Neonatal glucose level: ____(mmol/L) (number field from 0 – 20.0)

**16.** Infant on hypoglycemia protocol? (check “yes” if less than 2.6 mmol/L)

 Yes

 No

**17.** Presence of neonatal jaundice requiring phototherapy?

 Yes

 No

**18.** Infant on late preterm infant protocol?

 Yes

 No

**19.** Infant admitted to NICU?

 Yes

 No

**If NO, go to 21**

**20.**

Admission date: dd-mm-yyyy

Admission time: (HH:MM)

Discharge or Transfer to other institution date: dd-mm-yyyy

Discharge time: (HH:MM)

**21**. Infant received (select all applicable):

 Caloric supplementation in milk

 Neonatal intravenous fluids or gavage feeding

 Neonatal oxygen supplementation

 Neonatal invasive respiratory support

 CPAP

 Endotracheal intubation

 Resuscitation at delivery

 room positive pressure ventilation

 continuous positive airway pressure

 Delayed cord clamping

**If Delayed cord clampling,** Indicate duration of cord clamping: ____ Min (number field)

 Phototherapy

 Antibiotics

 Other medications (free text)

**22**. Infant examination (select all applicable):

 Presence of major congenital anomaly, **if yes, fill out the protocol deviation form**

 Neonatal complications

 Neonatal respiratory distress syndrome or transient tachypnea of the newborn

 Pneumothorax

 Hypoglycemia requiring intravenous fluids or gavage

 Exposure to antibiotics in neonatal period

 Necrotizing enterocolitis

 Intracranial hemorrhage

 Other (text)

**23.** Discharge or Transfer to other institution:

Date : dd-mm-yyyy

Time: HH:MM

**INFANT MEDICAL FOLLOW UP**

**3 MONTH**

**Date**: dd-mm-yyyy

**General health information**

**Please copy here the information from your visit to the pediatrician you obtained during the 2-month vaccination**

**1.** What is your baby’s most recent weight?

- (number field) kilograms (kg)

or

- (number field) pounds (lbs) and (number field) ounces (oz)

Date this measurement was recorded: dd-mm-yyyy

**2.** What is your baby most recent length?

- (number field) in centimeters (cm)

or

- (number field) inches

Date this measurement was recorded: dd-mm-yyyy

**3.** What is your baby most recent head circumference?

- (number field) in centimeters (cm)

or

- (number field) inches

Date this measurement was recorded: dd-mm-yyyy

**4.** Who is following up on your baby’s health? (please check all that apply)

󠇃 Pediatrician

󠇃 Family care physician

󠇃 Nurse practitioner

󠇃 Midwife

󠇃 Other, specify: (text)

**5.** Did your baby have any health condition since birth?

󠇃 Yes

󠇃 No

**If NO, go to 9**

**6.** Please indicate which health problem your baby had or still has (Select all that apply):

󠇃 Infections (like a cold, pneumonia, gastroenteritis, sore throat) **If marked, show 6.1 and 6.2**

6.1 Please describe: (notes)

6.2 Was medication necessary?

󠇃 Yes

󠇃 No

**If yes**, please indicate :

Name: (text)

Dose: (text)

Frequency (once a day, etc.): (text)

+Add

󠇃 Allergies (to milk, to antibiotics, or developed asthma) **If marked, show 6.3 and 6.4**

6.3 Please describe (notes)

6.4 Was medication necessary?

󠇃 Yes

󠇃 No

**If yes**, please indicate :

Name: (text)

Dose: (text)

Frequency (once a day, etc.): (text)

+Add

󠇃Other **If marked, show 6.5 and 6.6**

6.5 Please describe (notes)

6.6 Was medication necessary?

󠇃 Yes

󠇃 No

**If yes**, please indicate :

Name: (text)

Dose: (text)

Frequency (once a day, etc.): (text)

+Add

**7.** Was your baby hospitalized for any reason?

󠇃 Yes

󠇃 No

**If yes**, please explain the reason (notes)

**If yes**, When was your baby hospitalized?

From**:** dd-mm-yyyy

To : dd-mm-yyyy

**8.** Did your baby have a hearing screen?

󠇃 Yes

󠇃 No

**If Yes**, **show 8.1**

8.1 Do you know the result?

󠇃 Yes. Please explain: (notes)

󠇃 No

**9.** Does your baby stay with other people (i.e. other than yourself or the baby’s other parent) during the day? (for example, at daycare, at home with a baby sitter, at home with a grand parent)

󠇃 Yes. Please explain: (notes)

󠇃 No

**10**. Does your baby have any siblings?

󠇃 Yes

󠇃 No

**If yes, show 10.1**

10.1. Please tell us

󠇃 How many? (text)

󠇃 What are their ages? (text)

󠇃 Do they all live together? (notes)

󠇃 Do any of your baby’s siblings has a health condition? Please specify (notes)

**11.** When was your baby last vaccinated? dd-mm-yyyy

**12.** For which vaccines?

󠇃DPT-Polio-Hib

󠇃DPT-Polio

󠇃DPTP-Hib

󠇃MMR

󠇃Rotavirus (against diarrhea)

󠇃Varicella

󠇃Meningitis B

󠇃Hepatitis A

󠇃Hepatitis B (for example “Engerix”)

󠇃Varicella Vaccine (Chickenpox)

󠇃Pneumococcal Vaccine (Pneumonia, examples are Synflorix and Prevnar)

󠇃Other: Please specify: (text)

**INFANT MEDICAL FOLLOW UP**

**6 MONTH**

**Please copy here the information from your visit to the pediatrician you obtained during the 4-month vaccination**

**1.** What is your baby’s most recent weight?

- (number field) kilograms (kg)

or

- (number field) pounds (lbs) and (number field) ounces (oz)

Date this measurement was recorded: dd-mm-yyyy

**2.** What is your baby most recent length?

- (number field) in centimeters (cm)

or

- (number field) inches

Date this measurement was recorded: dd-mm-yyyy

**3.** What is your baby most recent head circumference?

- (number field) in centimeters (cm)

or

- (number field) inches

Date this measurement was recorded: dd-mm-yyyy

**4.** Is your baby’s health followed up by the same health caregiver?

󠇃 Yes

󠇃 No

**If NO**, why did you change? (notes)

**If NO,** who is your baby’s current caregiver?

(please check all that apply)

󠇃 Pediatrician

󠇃 Family care physician

󠇃 Nurse practitioner

󠇃 Midwife

󠇃 Other, specify: (text)

**5.** Did your baby have any health condition since last visit? (same choices)

󠇃 Yes

󠇃 No

**If NO, go to 9**

**6.** Please indicate which health problem your baby had or still has (Select all that apply):

󠇃 Infections (like a cold, pneumonia, gastroenteritis, sore throat) **If marked, show 6.1 and 6.2**

6.1 Please describe: (notes)

6.2 Was medication necessary?

󠇃 Yes

󠇃 No

**If yes**, please indicate :

Name: (text)

Dose: (text)

Frequency (once a day, etc.): (text)

+Add

󠇃 Allergies (to milk, to antibiotics, or developed asthma) **If marked, show 6.3 and 6.4**

6.3 Please describe (notes)

6.4 Was medication necessary?

󠇃 Yes

󠇃 No

**If yes**, please indicate :

Name: (text)

Dose: (text)

Frequency (once a day, etc.): (text)

+Add

󠇃Other **If marked, show 6.5 and 6.6**

6.5 Please describe (notes)

6.6 Was medication necessary?

󠇃 Yes

󠇃 No

**If yes**, please indicate :

Name: (text)

Dose: (text)

Frequency (once a day, etc.): (text)

+Add

**7.** Was your baby hospitalized for any reason since last visit?

󠇃 Yes

󠇃 No

**If yes**, please explain the reason (notes)

**If yes**, When was your baby hospitalized?

From**:** dd-mm-yyyy

To : dd-mm-yyyy

8. If your baby has siblings, was there it any change to the health of your baby’s sibling(s) since last visit?

- No siblings
- No change in any of the sibling’s health status since last visit
- Changes in sibling’s health status. Please specify: (notes)

9. When was your baby last vaccinated? dd-mm-yyyy

10. For which vaccines?

󠇃DPT-Polio-Hib

󠇃DPT-Polio

󠇃DPTP-Hib

󠇃MMR

󠇃Rotavirus (against diarrhea)

󠇃Varicella

󠇃Meningitis B

󠇃Hepatitis A

󠇃Hepatitis B (for example “Engerix”)

󠇃Varicella Vaccine (Chickenpox)

󠇃Pneumococcal Vaccine (Pneumonia, examples are Synflorix and Prevnar)

󠇃Other: Please specify: (text)

**INFANT MEDICAL FOLLOW UP**

**12 MONTH**

**1.** What is your baby’s most recent weight?

- (number field) kilograms (kg)

or

- (number field) pounds (lbs) and (number field) ounces (oz)

Date this measurement was recorded: dd-mm-yyyy

**2.** What is your baby most recent length?

- (number field) in centimeters (cm)

or

- (number field) inches

Date this measurement was recorded: dd-mm-yyyy

**3.** What is your baby most recent head circumference?

- (number field) in centimeters (cm)

or

- (number field) inches

Date this measurement was recorded: dd-mm-yyyy

**4.** Is your baby’s health followed up by the same health caregiver?

󠇃 Yes

󠇃 No

**If NO**, why did you change? (notes)

**If NO,** who is your baby’s current caregiver?

(please check all that apply)

󠇃 Pediatrician

󠇃 Family care physician

󠇃 Nurse practitioner

󠇃 Midwife

󠇃 Other, specify: (text)

**5.** Did your baby have any health condition since last visit? (same choices)

󠇃 Yes

󠇃 No

**If NO, go to 9**

**6.** Please indicate which health problem your baby had or still has (Select all that apply):

󠇃 Infections (like a cold, pneumonia, gastroenteritis, sore throat) **If marked, show 6.1 and 6.2**

6.1 Please describe: (notes)

6.2 Was medication necessary?

󠇃 Yes

󠇃 No

**If yes**, please indicate :

Name: (text)

Dose: (text)

Frequency (once a day, etc.): (text)

+Add

󠇃 Allergies (to milk, to antibiotics, or developed asthma) **If marked, show 6.3 and 6.4**

6.3 Please describe (notes)

6.4 Was medication necessary?

󠇃 Yes

󠇃 No

**If yes**, please indicate :

Name: (text)

Dose: (text)

Frequency (once a day, etc.): (text)

+Add

󠇃Other **If marked, show 6.5 and 6.6**

6.5 Please describe (notes)

6.6 Was medication necessary?

󠇃 Yes

󠇃 No

**If yes**, please indicate :

Name: (text)

Dose: (text)

Frequency (once a day, etc.): (text)

+Add

**7.** Was your baby hospitalized for any reason since last visit?

󠇃 Yes

󠇃 No

**If yes**, please explain the reason (notes)

**If yes**, When was your baby hospitalized?

From**:** dd-mm-yyyy

To : dd-mm-yyyy

8. If your baby has siblings, was there it any change to the health of your baby’s sibling(s) since last visit?

- No siblings
- No change in any of the sibling’s health status since last visit
- Changes in sibling’s health status. Please specify: (notes)

9. When was your baby last vaccinated? dd-mm-yyyy

10. For which vaccines?

󠇃DPT-Polio-Hib

󠇃DPT-Polio

󠇃DPTP-Hib

󠇃MMR

󠇃Rotavirus (against diarrhea)

󠇃Varicella

󠇃Meningitis B

󠇃Hepatitis A

󠇃Hepatitis B (for example “Engerix”)

󠇃Varicella Vaccine (Chickenpox)

󠇃Pneumococcal Vaccine (Pneumonia, examples are Synflorix and Prevnar)

󠇃Other: Please specify: (text)
