# Supplementary material for: Study protocol for the sheMATTERS study (iMproving cArdiovascular healTh in new moThERS): a randomized behavioral trial assessing the effect of a self-efficacy enhancing breastfeeding intervention on postpartum blood pressure and breastfeeding continuation in women with hypertensive disorders of pregnancy
Source: BMC Pregnancy Childbirth. 2023 Jan 26;23:68. doi: 10.1186/s12884-022-05325-3 (PMC9878496; doi:10.1186/s12884-022-05325-3)
Supplement: Supplementary file 4 — Additional file 4: Appendix 1. Trial Registration Data Set. [file 12884_2022_5325_MOESM4_ESM.docx]

**Appendix 1**. Trial Registration Data Set

| **Data category** |  |
| --- | --- |
| Primary registry and trial identifying number | ClinicalTrials.gov  Identifier: NCT04580927 |
| Date of registration in primary registry | October 9 2020 |
| Secondary identifying numbers |  |
| Source(s) of monetary or material support | Heart and Stroke Foundation  Rosenfeld Fund through McGill University  MUHC Department of Nursing  Fonds de Recherche du Québec – Santé |
| Primary sponsor | Research Institute of the McGill University Health Center  Dr C Karatzas  The Research Institute of the McGill University Health Centre 2155, rue Guy, 5e étage Montreal, QC H3H 2R9 |
| Secondary sponsor(s) |  |
| Contact for public inquiries | Natalie Dayan |
| Contact for scientific inquiries | Natalie Dayan |
| Public title | sheMATTERS |
| Scientific title | iMproving cArdiovascular healTh in new moThERS: “she MATTERS” |
| Countries of recruitment | Canada |
| Health condition(s) or problem(s) studied | - Hypertensive Disorder of Pregnancy - Pregnancy Complications - Pre-Eclampsia - Hypertension - Pregnancy-Induced Breastfeeding |
| Intervention(s) | Behavioral: breastfeeding self-efficacy |
| Key inclusion and exclusion criteria | Inclusion Criteria:   - Age >18 years. - Singleton live birth delivered at >34 weeks gestation. - Mother intends to breastfeed & initiated before postpartum hospital discharge (randomized portion) - Participant speaks and understands English or French. - Participant has a valid Medicare card - Participant has access to a cellular telephone and internet - Be available to attend in-person visits if COVID restrictions allow - Meet criteria related to the diagnosis of HDP |
|  | Exclusion criteria from randomized trial portion of the study:   - Maternal absolute contraindication to breastfeeding or condition that interferes with breastfeeding - Neonatal absolute contraindication to breastfeeding or condition that interferes with breastfeeding   Exclusion criteria from randomized OR observational portion of the study   - Infant born before 34 weeks gestation. - Maternal intensive care unit (ICU) admission lasting >24 hours. - Severe or uncontrolled psychiatric illness in the mother - Mother has active COVID-19 infection at time of postpartum hospitalization. - Previous BP-MOM participant (ISRCTN85493925, www.isrctn.com) |
| Study type | Interventional |
| Date of first enrollment | April 1, 2021 |
| Target sample size | 323 |
| Recruitment status | Recruiting |
| Protocol version | Protocol amendment number: 4  Issue date: May 2, 2022  Authors: ND, SS |
| Primary outcome(s) | 1. Evaluate whether a nurse-led BSE intervention will result in a lower systolic and/or diastolic BP 12 months postpartum 2. Evaluate whether a nurse-led BSE intervention will result in a lower need for antihypertensive therapy |
| Secondary outcome(s) | 1. Evaluate whether a nurse-led BSE intervention will result in longer duration of exclusive breastfeeding 2. Evaluate whether a nurse-led BSE intervention will result in higher rates of any continued breastfeeding at 6 months 3. Evaluate whether a nurse-led BSE intervention will result in lower metabolic syndrome |
