# Supplementary material for: Study protocol for the sheMATTERS study (iMproving cArdiovascular healTh in new moThERS): a randomized behavioral trial assessing the effect of a self-efficacy enhancing breastfeeding intervention on postpartum blood pressure and breastfeeding continuation in women with hypertensive disorders of pregnancy
Source: BMC Pregnancy Childbirth. 2023 Jan 26;23:68. doi: 10.1186/s12884-022-05325-3 (PMC9878496; doi:10.1186/s12884-022-05325-3)
Supplement: Supplementary file 5 — Additional file 5: Appendix 2. sheMATTERS Informed Consent Form. [file 12884_2022_5325_MOESM5_ESM.docx]

Appendix 2. sheMATTERS Informed Consent Form

**INFORMATION AND CONSENT FORM**

| **Research Study Title:** | i**M**proving c**A**rdiovascular heal**T**h in new mo**T**h**ERS**: “**she MATTERS”** |
| --- | --- |
| **Protocol number:** | MP-37-2021-7201 |
| **Researchers responsible for the research study:** | **Dr. Natalie Dayan**  McGill University Health Centre Research Institute  Centre for Outcomes Research and Evaluation (CORE)  5252 de Maisonneuve West, 2B.40  Montréal QC H4A 3S5  **Dr. Sonia Semenic, N, PhD**  Ingram School of Nursing, McGill University  Nurse Scientist, McGill University Health Center  680 Sherbrooke Street West, Montreal, QC, H3A 2M7  Montreal, QC H3A 2M7 |
| **Co-Investigator(s)/sites:**  **Co-Investigators at McGill**  **University**  **Co-Investigator at Toronto University** | **Dr. Atanas Nedelchev**  St. Mary’s Hospital Center  3830 Lacombe Avenue Montreal, QC H3T 1M5  **Dr. Graeme Smith**  Kingston General Hospital  76 Stuart Street Kingston, Ontario K7L 2V7  **Dr. Suhad Ali, PhD**  **Dr. Deborah Da Costa, PhD**  **Dr. Richard Brown, MD**  **Dr. Tuong-Vi Nguyen, MD**  **Dr. Cindy-Lee Dennis, PhD** |
| **Funders:** | Heart and Stroke Foundation of Canada  McGill University  Rosenfeld Heart Foundation |

**INTRODUCTION**

You are being invited to take part in a research study that examines breastfeeding and maternal health outcomes in women who have had high blood pressure or preeclampsia in pregnancy. You are being invited to participate in this study because you have been diagnosed with one of these conditions.

However, before you accept to take part in this study and sign this information and consent form, please take the time to read, understand and carefully examine the following information. You may also want to discuss this study with your family doctor, a family member or a close friend.

This form may contain words that you do not understand. We invite you to speak to the researcher responsible for this study (the “study doctor”) or to other members of the research team, and ask them to explain to you any word or information that is unclear to you before you sign this form.

**BACKGROUND**

Breastfeeding may lower blood pressure and improve cardiovascular risk factors in the mother. Interventions designed to increase women’s confidence about breastfeeding have been effective in improving breastfeeding practices in healthy postpartum women (i.e., after childbirth), but it is not known whether these types of interventions are helpful for women who have had preeclampsia or high blood pressure in pregnancy. This is an important question to answer, since such women may derive substantial benefits from breastfeeding.

A proper diet favors babies’ healthy growth. Breastmilk provides optimal nutrition for children during their first six months of life, which may improve children's growth.

In this study of women who have had high blood pressure or preeclampsia in pregnancy, we will be testing a nurse-led intervention designed to improve women’s confidence about breastfeeding. An independent part of this study (a sub-study) will look at how different ways of feeding influences the growth of babies whose mothers had hypertension during pregnancy.

This research study is called multi-site open-label, randomized control trial. It means that it will be conducted in different places at the same time (multi-site). It is called control trial because it will evaluate a clinical procedure, in this case a breastfeeding support intervention, between participants receiving the intervention and participants not receiving it. Open-label means that you, the study doctor, and the research team will know which study group will be assigned to you. Randomized means that the study group you may be in will be decided by chance, like a flip of a coin.

**PURPOSE OF THE RESEARCH STUDY**

The purpose of this study is to assess a breastfeeding support intervention (conducted one-on-one by a nurse) in women who had blood pressure problems during pregnancy. We will examine whether this intervention improves breastfeeding practices and whether this lowers blood pressure in the postpartum period. In the long-term, by reviewing medical charts we will see whether breastfeeding helps to lower the chance of being hospitalized with heart disease or stroke and / or the need of medication for heart conditions up to 15 years after delivery.

We would like to follow up the babies of mothers with hypertension in the first 12 months of life by collecting some information about their growth and development. This part of the study is independent of the mother’s participation.

For this research study, we will recruit approximately 278 breastfeeding women and 45 non-breastfeeding women, aged 18 years old or older, who had high blood pressure in pregnancy, from three participating centers.

In Quebec there will be two centers: (1) the McGill University Health Centre-Royal Victoria Hospital (MUHC-RVH) and (2) St. Mary’s Hospital Centre (SMHC) and the third center will be in Ontario at the (3) Kingston General Hospital (KGH).

**DESCRIPTION OF THE RESEARCH PROCEDURES**

**1. Duration and number of visits**

Your participation in this research study will last 12 months and will include completion of online questionnaires at five (5) time points, as well as two (2) in-person clinical follow-up visits at 6 months and 12 months postpartum (after the birth of the baby). Because of the coronavirus-19 (COVID-19) pandemic, we may adapt these in-person visits to include telephone or video-conference options. Participants that will be receiving the nurse-led breastfeeding intervention will also have weekly breastfeeding support sessions by telephone for the first 6 weeks after they left the hospital, a phone or videoconference session at 3 months postpartum, and can contact the research nurse by phone or text as needed for up to 6 months postpartum. The telephone contacts and/or clinic visits will last 30-60 minutes for all participants.

**2. Study participation**

When participating in this research study, if you are planning on breastfeeding you will be assigned to one of the following groups:

**Group 1-Intervention breastfeeding women**:

If you are assigned to Group 1, you will receive usual postpartum care plus an additional nurse-led breastfeeding support intervention.

**Group 2-Control breastfeeding women**:

If you are assigned to Group 2, you will receive usual postpartum care only.

Among breastfeeding women, this study is randomized which means that breastfeeding mothers will be assigned by chance (like a flip of a coin) to one of the groups. You may not choose the group to which you will be assigned. One person out of two (50%) will receive the study intervention (Group 1) whereas one person out of two (50%) will receive standard postpartum care (Group 2).

**Group 3-Observational (non-breastfeeding women)**

As a non-breastfeeding participant, you will receive usual postpartum care that does not include breastfeeding support.

No matter in which group you are participating, you are also invited to provide information about your baby in the first 12 months of his/her life.

All participating mothers, breastfeeding or not, will have access to a private study email address to communicate with the research team for any reason, at any time, during your 12-month study period. The private email address is [shematters@rimuhc.ca](mailto:shematters@rimuhc.ca). Access to this email address is restricted to the team members only.

**3. Tests and procedures**

Study follow-up visits:

The study tests and procedures described below occur at the following time:

- at the time you start the study (about at the time of birth),
- 6 weeks after birth,
- 3 months after birth,
- 6 months after birth, and
- 12 months after birth.

Most of these study follow-up visits will occur **remotely**, that is from home from your smartphone or computer, without the need to displace yourself.

General description of tests and procedures of study protocol: Your participation in this study includes completion of study questionnaires, blood pressure, weight and measurements of your waist and hips, routine blood tests, and review of your and your baby’s medical files. A member of the research team will collect health information including a review of your medical chart, measures of your blood pressure and weight, and information gathered through questionnaires that you will be asked to complete (for example about breastfeeding, feelings of anxiety or sadness, your eating and exercise habits).

The routine blood tests include cholesterol profile, blood sugar levels, and evaluation of kidney function and will be done at 6 months postpartum, and at 12 months postpartum. At baseline information of available tests results will be obtain from your medical chart.

Biomarker analysis: At the start of the study and at 12 months postpartum we will collect additional blood for biomarker analysis. A biomarker is a substance that can be measured in the blood and is a sign of a normal or abnormal process, or a condition or disease. Biomarker analysis help researcher to test and learn about long-term blood vessel response to high blood pressure in pregnancy.

As much as possible, blood draws for biomarker analysis will occur at the same time as we obtain blood for routine tests. This may change with the COVID-19 pandemic and you will be informed. There is a chance that an additional blood draw may be required to complete this step of the study.

Health record linkage: Because the researchers conducting this study would like to learn if and how breastfeeding affects the cardiovascular system as women get older, we will also ask if you agree that we keep your health insurance number so that we can track hospitalizations, surgeries and the medicines you may be prescribed during the next 15 years after completion of the study. This data is all linked electronically to your health insurance number (RAMQ number). This health record linkage is independent of the rest of the study, so you may actively participate during the 12 months but not agree to have us track your long-term health information, OR you may agree ONLY to have us track your information using your health insurance number and NOT actively participate in the 12-month study. If you agree to participate in the Linkage arm only we will also ask for a small blood sample of 5 ml (half table spoon) coordinated with any blood tests that are done in hospital as part of your routine care.

Home blood pressure measurement: An important part of the study is blood pressure measurement which will be done both at home using a machine that will be loaned to you, and by trained personnel. The research team will loan you a blood pressure machine when you start the study. The machine will have to be returned in between study visits and returned to you as needed, and the researcher team will pay the shipping costs. The researchers conducting this study will help you upload a smartphone or web software application (free of charge) which will help the team receive your blood pressure values during the scheduled study follow-up visits.

The following table describes the tests and procedures throughout the study depending on which group you are assigned to. Please note that at any time during your participation, you may complete the questionnaires in more than one sitting if you wish.

| **DESCRIPTION OF STUDY PROCEDURES** | | |  |
| --- | --- | --- | --- |
| **During the study** | **Group 1 Intervention** | **Group 2**  **Control** | **Group 3**  **Non-breastfeeding** |
| **24-48 hours after giving birth in the hospital**: | **X** |  |  |
| Two in-hospital face-to-face* breastfeeding session with the study nurse (lasting about 30 minutes each). |  |  |  |
| Completing questionnaires about your baby’s feeding and your breastfeeding confidence (5 minutes). | **X** | **X** |  |
| Completing questionnaires about your age, years of schooling, social situation, general health, obstetrical history, mood (feeling happy, depressed or anxious), and diet (30-45 minutes). | **X** | **X** | **X** |
| A nurse will measure your height, weight, and blood pressure. | **X** | **X** | **X** |
| Approximately 4 ml of blood (one teaspoon) will be collected to test for proteins related to inflammation and heart function (“Biomarker Analysis”), which will be coordinated with any blood tests that are done in hospital as part of your routine care. | **X** | **X** | **X** |
| You will be provided with a home Blood Pressure machine (A&D medical device) and instructions for use. You will also be instructed on how to download and use the Sphygmo^TM^ Application**, and will be given a measuring tape and shown how to obtain your hip and waist circumference. | **X** | **X** | **X** |
| **From time of leaving the hospital**  **to 6 weeks after leaving the hospital:** | **X** |  |  |
| Breastfeeding support sessions by telephone with the study nurse lasting about 30 minutes each, **once a week** for the **first 6 weeks** after leaving the hospital. A private/secure online session with camera communication may be arranged if needed. |  |  |  |
| The study nurse will also be available by phone/text in case you need additional breastfeeding support, for up to 6 months after leaving the hospital. | **X** |  |  |
| **6 weeks after leaving the hospital:** | **X** | **X** |  |
| Completing questionnaires about your baby’s feeding and your breastfeeding confidence (10 minutes). |  |  |  |
| Measuring your Blood Pressure at home using the A&D device provided to you and transmitting the values via the application that will be installed on your cellular phone to the research nurse. | **X** | **X** | **X** |
| Completing questionnaire about your mood (feeling happy, depressed or anxious) (5-10 minutes). | **X** | **X** | **X** |
| **3 months after leaving the hospital:** | **X** |  |  |
| Completing a questionnaire about your satisfaction with the breastfeeding intervention (5 minutes). |  |  |  |
| One breastfeeding “booster” support session (via telephone or secured videoconference) with the study nurse, lasting about 30 minutes. | **X** |  |  |
| Completing questionnaires about your baby’s feeding and your breastfeeding confidence (10 minutes). | **X** | **X** |  |
| Completing questionnaires about your mood, diet, physical activity, and any new medical diagnoses and medications that you use (30 minutes). | **X** | **X** | **X** |
| You will take your Blood Pressure measurement as explained above. | **X** | **X** | **X** |
| Complete information related to your baby’s health (if you accept to participate in this part of the study) | **X** | **X** | **X** |
| **6 months after leaving the hospital:** | **X** |  |  |
| Completing a questionnaire about your satisfaction with the breastfeeding intervention (5 minutes). |  |  |  |
| Completing questionnaires about your baby’s feeding and your breastfeeding confidence (10 minutes). | **X** | **X** |  |
| You will take your Blood Pressure measurement at home as explained above. | **X** | **X** | **X** |
| Completing questionnaires about your mood, diet, physical activity, and any new medical diagnoses and medications that you use (30 minutes). | **X** | **X** | **X** |
| You will visit your doctor at the hospital* and a nurse will measure your weight, waist and hip circumference, and blood pressure. | **X** | **X** | **X** |
| Approximately 4 ml (1 teaspoon) of blood will be collected for tests including cholesterol, glucose, protein in the urine and kidney function. | **X** | **X** | **X** |
| Complete information related to your baby’s health (if you accept to participate in this part of the study) | **X** | **X** | **X** |
| **12 months after leaving the hospital:** | **X** | **X** |  |
| Completing questionnaires about your baby’s feeding your breastfeeding confidence (10 minutes). |  |  |  |
| Completing questionnaires about your mood, diet, physical activity, and any new medical diagnoses and medications that you use (30 minutes). | **X** | **X** | **X** |
| You will take your Blood Pressure measurement at home as explained above. | **X** | **X** | **X** |
| You will visit your doctor at the hospital* and a nurse will measure your weight, waist and hip circumference, and blood pressure. | **X** | **X** | **X** |
| Approximately 4 ml (1 teaspoon) of blood will be collected for cholesterol, glucose, protein in the urine, and kidney function, and an additional 4 ml will be collected at the same time to test for “Biomarkers”. | **X** | **X** | **X** |
| Complete information related to your baby’s health and your baby’s activities like playing, sitting, eating (if you accept to participate in this part of the study) | **X** | **X** | **X** |
| **Following the 12-month visit and up to 15 years:** | **X** | **X** | **X** |
| Long-term follow-up where you may authorize the research team to have access to your medical records (emergency visits, hospitalizations and medications) for up to 15 years (nothing additional will be asked of you). |  |  |  |

*All in-person visits may be adapted due to the COVID-19 pandemic to be by phone or videoconference using secured connections.

**The Sphygmo^TM^ application will be installed on your cellular phone for the duration of the study (12 months) and it will be up to you if you want to continue to use the application after the completion of the study or if you would like to uninstall it. The application is free of charge and available to anyone via Google Play or the Apple Store.

You will be given an email address to use only with the Sphygmo^TM^ application which will not identify you.

You will find information on privacy and confidentiality of the collected data using this application below.

At the end of this document, you will be asked about your interest to participate in other research studies in the future. You are free to accept or refuse to be contacted in the future and you can change your mind at any time without consequences.

**BENEFITS ASSOCIATED WITH THE RESEARCH STUDY**

You may or may not personally benefit from your participation in this research study. However, the results from this study may benefit the future postpartum care and breastfeeding support for women who have high blood pressure during pregnancy or preeclampsia.

**RISKS ASSOCIATED WITH THE RESEARCH STUDY**

This study involves some additional blood draws that will be collected (as much as possible) at the same time as the blood draws that are done routinely. Therefore, there will not be a need for additional puncture. Blood draws may cause temporary discomfort, minor bleeding, light-headedness, and rarely, fainting.

You will be completing questionnaires that may feel long and tiring. You can complete them at different sittings. If at any time you feel sad or anxious you can contact the research team (phones below) for immediate referral to get appropriate help.

In addition, there are risks associated with any loss of confidentiality of your and / or your child (if you participate in this part of the study) health information. To minimise this risk, your personal information and the one of your baby will be coded. However, absolute confidentiality can never be guaranteed. We will take special precautions to ensure confidentiality of all information including removing any identifying information and reporting the results in aggregate form only, as described in the section Confidentiality below

**OTHER POSSIBLE TREATMENTS**

This study does not provide any medical treatment. If you decide to breastfeed and do not wish to participate in this study, you will get the usual support for breastfeeding available in your hospital and community. The treating physician will discuss all alternatives with you. This study will not intervene in the treatment or the decisions made by your treating physician.

**VOLUNTARY PARTICIPATION AND THE RIGHT TO WITHDRAW**

Your participation in this research study is voluntary. Therefore, you may refuse to participate. You may also withdraw from the study at any time, without giving any reason, by informing the study doctor or a member of the research team.

Your decision not to participate in the study, or to withdraw from it, will have no impact on the quality of care and services to which you are otherwise entitled, or on your relationship with the study doctor or clinical team.

The study doctor, the Research Ethics Board, or the funding agency may put an end to your participation without your consent. This may happen if new findings or information indicate that participation is no longer in your interest, if you do not follow study instructions, or if there are administrative reasons to terminate the study.

If you choose to withdraw from the study, all study data and blood samples collected will be destroyed within two business days of withdrawal. If at the time you choose to withdraw study results have already been published or presented, we will be unable to withdraw your data from these publications and presentations. However, your data would be excluded from any further publications or presentations.

Any new findings that could influence your decision to stay in the research study will be shared with you as soon as possible.

**CONFIDENTIALITY**

During your participation in this study, the study doctor and research team will collect and record information about you (and your baby if you participate in this part of the study) in a study file. They will only collect information required to meet the scientific goals of the study.

The study file may include information from your medical chart, including your identity, concerning your past and present state of health, your lifestyle habits, as well as the results of the tests, exams, and procedures that you will undergo during this research study. The study will also include information about your baby at the time the baby was born and if you agree to participate, it will also include some health information about your baby during the 12 month of your participation in the study. Your research file could also contain other information, such as your name, sex, date of birth and ethnic origin.

All the information collected during the research study will remain strictly confidential to the extent provided by law. You will only be identified by a code number. The key to the code linking your name to your study file will be kept by the study doctor.

The information collected online will be managed through REDCap system hosted in Montreal complying with Canadian privacy laws.

The data collected during the study will be coded and stored by the study doctor (Dr. Natalie Dayan) at the RIMUHC for 25 years for the exclusive objectives of this study and then destroyed. The information that is collected for this study will be kept in a locked and secure area. Only the principal investigator will be able to link the code to your personal information.

Blood samples taken for biomarker analysis will be coded, processed and stored in Dr. Suhad Ali’s lab (co-investigator with Drs. Dayan and Semenic for this study). Your identity will not be disclosed to those analyzing your blood samples.

To ensure your safety, a copy of this information and consent form and blood test results will be placed in your medical chart. As a result, any person or company to whom you give access to your medical chart will have access to this information.

All participating mothers will have access to a private study email address to communicate with the research team. Access to the messages sent to this email address ([shematters@rimuhc.ca](mailto:shematters@rimuhc.ca)) is restricted to the team members only.

The application (Sphygmo ^TM^) that will be installed in your phone follows Canadian privacy laws. All information is encrypted on the app and in the server. The data is housed in a server located in Ontario with a company that specializes in medical data storage (Server Cloud Canada). Only the Chief Security Officer of the company (which is called “mmHg”) has access to the data, necessary for maintenance and management of the platform. Otherwise only researchers conducting this study will have access to the information. You will be given a user name and password to use only with the Sphygmo^TM^ application which will not identify you.

The data may be published or shared during scientific meetings; however, it will not be possible to identify you.

For monitoring, control, safety, and security, your study file as well as your medical charts may be examined by a person mandated by Canadian or international regulatory authorities, such as Health Canada, as well as by representatives of the institution, or the Research Ethics Board. All these individuals and organizations adhere to policies on confidentiality.

You have the right to consult your study file in order to verify the information gathered, and to have it corrected if necessary. However, in order to protect the scientific integrity of the research study, accessing certain information before the study is ended may require that you be withdrawn from the study.

**INCIDENTAL FINDINGS**

Material incidental findings are findings made during the study that may have significant impacts on your current or future wellbeing or that of your family members. A material incidental finding concerning you in the course of this research will be communicated to you and to a health professional of your choice.

**FUNDING OF THE RESEARCH STUDY**

The study doctor and the institution have received funding from the Heart and Stroke Foundation of Canada, McGill University and Rosenfeld Foundation for the completion of the research study.

**COMPENSATION**

You will be compensated for parking or transportation expenses an amount of $10.00 per in-person study visit, for a total of two (2) visits. A Gift Bag valued of $20.00 CDN, containing samples of newborn products from collaborating suppliers will be given to you immediately after informed consent has been obtained.

At the end of the study, you will receive a gift card (value $20) as a token of appreciation for completing the study.

If you withdraw from the study, or are withdrawn before it is completed, you will receive compensation proportional to the number of visits you have completed (for example, if you complete half of the study which is the same as 6 months participation you will receive a $10 gift card).

**SHOULD YOU SUFFER ANY HARM**

Should you suffer harm of any kind following any procedure related to the research study, you will receive the appropriate care and services required by your state of health.

By agreeing to participate in this research study, you are not waiving any of your legal rights nor discharging the study doctor, the sponsor or the institution, of their civil and professional responsibilities.

**CLINICAL TRIAL REGISTRATION**

A description of this clinical trial will be available on [http://www.ClinicalTrials.gov](http://www.ClinicalTrials.gov/). This Website will not include information that can identify you. At most, the Website will include a summary of the results. You can search this Website at any moment. The sheMATTERS project is registered with the number NCT04580927.

**CONTACT INFORMATION**

If you have questions or if you have a problem you think may be related to your participation in this research study, or if you would like to withdraw, you may communicate with the study doctor or with someone on the research team at the following number:

At McGill University Health Center (MUHC):

Dr. Natalie Dayan, MD MSc

E-Mail: natalie.dayan@mcgill.ca

Tel #: 514-934-1934 x 76125

Dr. Sonia Semenic, N, PhD

E-Mail: sonia.semenic@mcgill.ca

514-398-1281

At St. Mary’s Hospital Center:

Dr. Atanas Nedelchev

E-Mail: helene.weibel@mcgill.ca

Tel #: 514-345-3511 ext. 5133

Project Manager: Iris Groisman, PhD

E-Mail: [Iris.groisman@rimuhc.ca](mailto:Iris.groisman@rimuhc.ca)

Tel: 514-934-1934 x 76147

For any question concerning your rights as a research participant taking part in this study or if you have comments, or wish to file a complaint, you may communicate with the Patient Ombudsman:

At MUHC: [ombudsman@muhc.mcgill.ca](mailto:ombudsman@muhc.mcgill.ca); or by phone at 514 934 1934 ext. 48306

At St Mary’s Hospital Center: [commissariat.plaintes.comtl@ssss.gouv.qc.ca](mailto:commissariat.plaintes.comtl@ssss.gouv.qc.ca); or by phone at 1-844-630-5125

**OVERVIEW OF ETHICAL ASPECTS OF THE RESEARCH**

The McGill University Health Centre Research Ethics Board reviewed this study and is responsible for monitoring it at all participating institutions in the health and social services network in Quebec.

| **Research Study Title:** | i**M**proving c**A**rdiovascular heal**T**h in new mo**T**h**ERS**: “**she MATTERS”** |
| --- | --- |

**SIGNATURES**

***Signature of the participant***

I have reviewed the information and consent form. Both the research study and the information and consent form were explained to me. My questions were answered, and I was given sufficient time to make a decision. After reflection, I consent to participate in this research study in accordance with the conditions stated above. By **checking (√) Yes** below I consent to partake in the following research options:

| Yes  No | I consent to participate in the 12-months study |
| --- | --- |
| Yes  No | I consent to participate in the long-term follow-study where I authorize the research team to have access to all my medical records for up to 15 years and nothing additional will be asked of me. |
| Yes  No | I consent to complete (some) questionnaires about my baby’s health and growth |
| Yes  No | I authorize the researchers in charge of this study to communicate with me directly to ask if I am interested in participating in another research study. If I accept, my phone number and email will be collected on a separate document |

**Name of Participant Signature Date**

***Signature of the person obtaining consent***

I have explained the research study and the terms of this information and consent form to the research participant, and I answered all his/her questions.

**Name of the person obtaining consent Signature Date**

***Commitment of the principal investigator***

I certify that this information and consent form were explained to the research participant, and that the questions the participant had were answered.

I undertake, together with the research team, to respect what was agreed upon in the information and consent form, and to give a signed and dated copy of this form to the research participant.

**Name of the principal investigator Signature Date**
