# Supplementary material for: Study protocol for the sheMATTERS study (iMproving cArdiovascular healTh in new moThERS): a randomized behavioral trial assessing the effect of a self-efficacy enhancing breastfeeding intervention on postpartum blood pressure and breastfeeding continuation in women with hypertensive disorders of pregnancy
Source: BMC Pregnancy Childbirth. 2023 Jan 26;23:68. doi: 10.1186/s12884-022-05325-3 (PMC9878496; doi:10.1186/s12884-022-05325-3)
Supplement: Supplementary file 6 — Additional file 6: Appendix 3. Management of Biological Specimens. [file 12884_2022_5325_MOESM6_ESM.docx]

Appendix 3. Management of Biological Specimens

1. **PURPOSE**

This Standard Operating Procedure (SOP) describes the management of biological specimens obtained from clinical research subjects. Processes include collection, processing, storage, and handling from collection to destruction, within the institution.

1. **SCOPE**

This SOP applies to sheMATTERS undertaken at all three sites (RI MUHC, SMH, JGH, and KGH), and to those clinical research personnel responsible for biological specimen handling namely the Project Manager and Coordinator, Research Assistants, Research Nurses and Master students involved in the project.

1. **RESPONSIBILITIES**

The Investigators are responsible for ensuring that the specimen handling processes meet all of the applicable regulatory, International Conference on Harmonisation (ICH) Good Clinical Practice (GCP), sponsor, and local requirements.

Any or all parts of this procedure may be delegated to appropriately trained study team

members, but remain the ultimate responsibility of the Investigators.

1. **PROCEDURES**
   1. Blood collection for study purposes occurs at three time points: baseline, 6- and 12-month visit. If the participant consented to long-term linkage and not the active study, then blood collection will occur at baseline only.

- At baseline, about 4 ml of blood are obtained for biomarker analysis.
- At 6- and 12-month visits the blood draw purpose is to conduct routine tests.
- In addition, at 12-month visit about 4 ml of blood will be obtain when routine tests are conducted for biomarker analysis.

- 1. Collection of blood at baseline- Samples for Biomarker Study:

Supplies at the site (Royal Victoria Hospital / RI-MUHC; SMH; JGH):

- - Blood collection tubes (Vacutainer tubes Becton Dickinson BD SST™ Tubes with Gel and Clot Activator, red caps)
  - Biohazard bags
  - Blood requisition for an additional 4 ml blood draw to be collected in the indicated red cap tube
  - Lick-proof containers for sample transport
  - KGH will be supplied with same type of tubes and will follow its own procedure for sample handling and transport

Procedure for collecting blood at baseline:


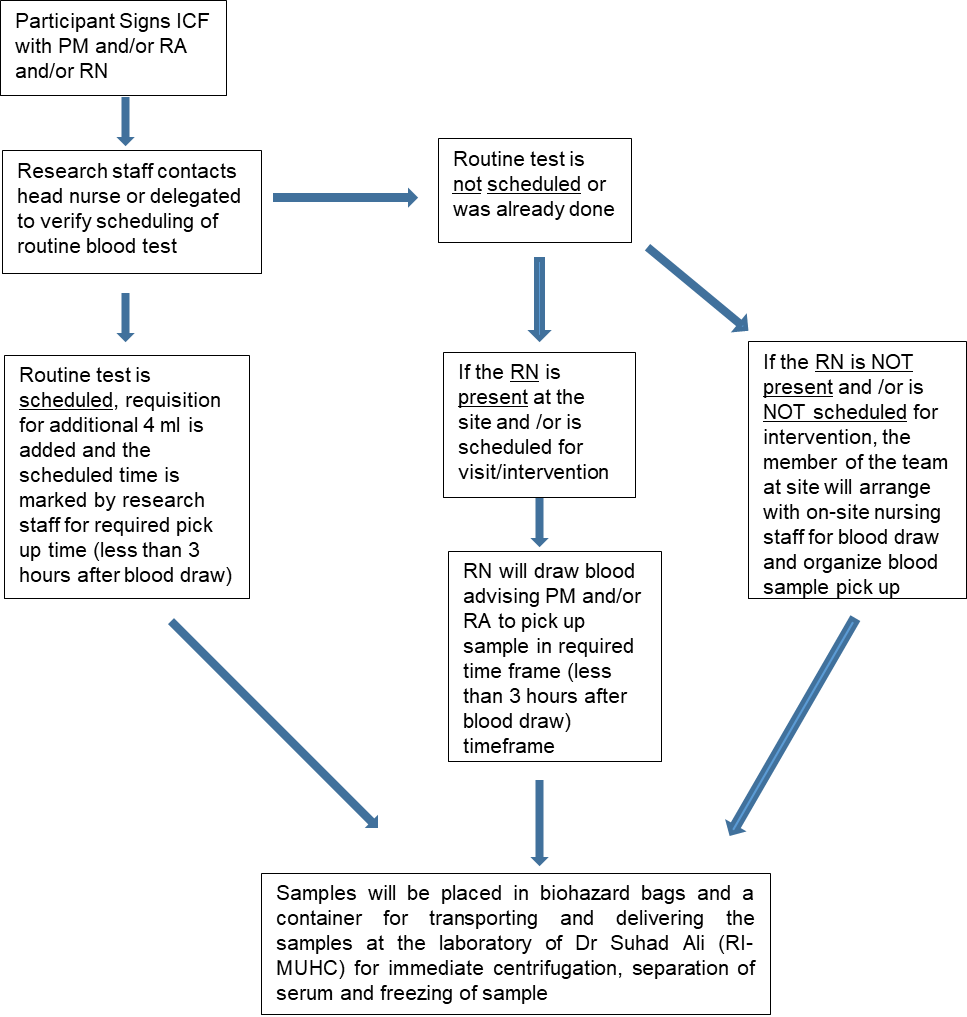


- Samples will be placed in biohazard bags and in a container for transporting and delivering the samples to the laboratory of Dr Suhad Ali for immediate centrifugation, separation of serum and freezing. This procedure has to take place within 3 hours of sample collection.
- Tubes will be coded at time of blood collection. If there are personal identifiers on the tube these will be covered and replaced by participants’ code.
- Vacutainer tubes (Becton Dickinson BD SST™ Tubes with Gel and Clot Activator) will be used to collect samples. Blood samples will be incubated for 60 min at room temperature to allow clotting and subsequently centrifuged at 1500 g for 15min. Serum will be collected and a second centrifugation will be performed on the serum at 2000 g for 10 min to clear it from any contaminating cells. Serum samples will be aliquoted and stored at -80 °C until use.
- Appropriate CRF (MCQ baseline) will be completed indicating if sample for biomarkers was obtained
  1. Blood tests at 6-months
- Standard of care tests will be pursued according to protocol. If on-site visits will be permitted, the staff will try to accommodate the blood tests at the same time as the medical consult
- Participants will be provided with a blood test requisition which could we used at participants’ preferred location instead or in case on-site visits are not recommended
  1. Blood tests at 12-months
- Standard of care tests will be pursued according to protocol. If on-site visits will be permitted, the staff will try to accommodate the blood tests at the same time as the medical consult to obtain sample for biomarker analysis at the same time
- Participants will be provided with a blood test requisition which could be used at participants’ preferred location instead or in case on-site visits are not recommended
- If the case be, a second blood draw will be obtained at participants’ 12-month on-site visit or at home if restrictions during the pandemic are lifted
  1. Storage
- Serum obtained at RI MUHC, SMH, and JGH will be stored at Dr Suhad Ali’s laboratory until processing
- Those processing serum samples will only manage de-identified (coded) samples. The key linking the code number and participants’ information will be handed by the Principal Investigators or delegated personnel
- Samples collected at KGH will be managed following the same processing protocol and will be shipped without identifiers to the laboratory of Dr. Ali at the end of the sample collection period
- Biological material will be used according to protocol
  1. Biological Specimens Destruction
- Samples will be stored according to protocol and destroyed afterwards following RI-MUHC processes

1. **ADDITIONAL RESPONSIBILITIES OF BIOLOGICAL SPECIMEN COLLECTION**

- Ensure that staff is qualified and trained on the study specific tasks delegated to them
- Assess the health and safety risks involved in the collection and analysis of the

biological specimens, as well as the precautionary measures required to prevent

accidents

- All laboratory abnormalities should be reported to the Investigators for assessment and all assessments (clinical significance) should be printed, initialed and dated by the Investigator (examples are power failures rendering serum unsuitable for further use)
- Make the safety information available to the persons coming in contact or handling the biological specimens and ensure that these persons receive training in the appropriate safety practices.
- Ensure the safety and well-being of subjects during the collection of specimens
- Laboratory records to retain include normal ranges for study tests and laboratory results/reports
- Document specimen collection is documented in the CMF Mother form in REDCap specifying the collected volume in ml
